# Large Enhancement of Spin-Flip Scattering Efficiency at Y_3_Fe_5_O_12_/Pt Interfaces Due to Vertical Confinement

**DOI:** 10.1021/acs.nanolett.5c05598

**Published:** 2026-05-13

**Authors:** Haripriya Madathil, Pranav Pradeep, Paul Noël, Saül Vélez

**Affiliations:** † Spintronics and Nanodevices Laboratory, Departamento de Física de la Materia Condensada and Condensed Matter Physics Center (IFIMAC), 16722 Universidad Autónoma de Madrid, Madrid E-28049, Spain; ‡ Université de Strasbourg, CNRS, IPCMS, UMR 7504, Strasbourg F-67034, France; § Instituto Nicolás Cabrera, Universidad Autónoma de Madrid, Madrid E-28049, Spain

**Keywords:** magnons, spin-flip scattering, harmonic transport, nonlinear magnetoresistance, spin Hall magnetoresistance

## Abstract

Magnons, the quanta of spin angular momentum, can be excited in magnetic insulators by spin-flip scattering processes induced by currents applied to a heavy-metal overlayer. The efficiency of generating nonequilibrium magnons is characterized by the interface spin current *j*_s_^int^, whose magnitude is considered to depend on the thermal magnon population. Here, we investigate nonlinear magnetoresistance phenomena in Pt arising from current-driven nonequilibrium magnons in Y_3_Fe_5_O_12_ (YIG). Remarkably, we find that spin-flip scattering processes are dominated by subthermal magnons at room temperature, resulting in a large modulation of *j*_s_^int^ with the magnetic field and YIG thickness. Concretely, reducing the YIG thickness from 100 to 10 nm increases *j*_s_^int^ by a factor ∼20, while increasing the magnetic field exponentially suppresses the magnon generation efficiency. These findings challenge the current understanding on *j*_s_^int^ and indicate that electrically driven magnonic effects such as damping compensation and magnon condensation can be largely boosted through device miniaturization.

## Graphical abstract

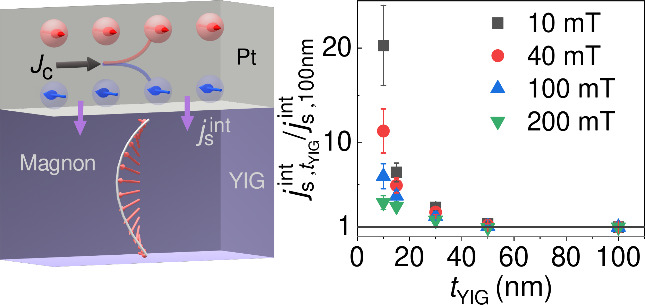

Magnetic insulators (MIs) interfaced with heavy metals (HMs) are an ideal platform for realizing magnonic devices operated by charge currents.^1–3^ Diffusive magnons, parametrized by the magnon chemical potential,^4^ are created in such heterostructures by interfacial spin-flip scattering processes. Most experiments to date have been devoted to investigating the transport characteristics of diffusive magnons by employing nonlocal lateral structures,^5–11^ whereas less attention has been given to exploring the MI/HM interface spin conductance and emerging phenomena at the metal side.^12,13^

Variations in the magnon population lead to changes in the magnetization. Thereby, any magnetoresistance that depends on the magnetization is expected to be modulated when magnons are created or annihilated by spin currents. Recently, Noël et al. showed that the creation/annihilation of magnons in Pt/Y_3_Fe_5_O_12_ (YIG) results in a nonlinear magnetoresistive response in Pt.^13^ However, the physical mechanisms governing the amplitude of this effect remain poorly understood.

In this work, we investigate nonlinear magnetoresistance arising from magnon creation/annihilation in YIG/Pt of varying YIG thickness and demonstrate that the effect is dominated by subthermal magnons at room temperature. As a result, the interface spin current *j*_s_^int^ is largely modulated by the magnetic field and YIG thickness. Remarkably, reducing the YIG thickness from 100 to 10 nm enhances the magnon modulation efficiency by a factor of ∼20, which we attribute to the vertical confinement of the subthermal magnon modes. These results evidence the potential to realize spin–orbit torque nano-oscillators, two-dimensional magnonic circuits, and explore magnon Bose–Einstein condensate physics at the nanoscale.

## Nonlinear Magnetoresistance Due to Magnon Creation/Annihilation

In Pt/YIG, the relevant magnetoresistance is the spin Hall magnetoresistance (SMR), which stems from the interaction of spin currents in Pt with magnetic moments in YIG.^14,15^ While the amplitude of the SMR depends on specific parameters of the materials defining the interface,^15^ the Hall response in ferro/ferrimagnets with in-plane magnetization **M** = (*M*_*x*_, *M*_*y*_) scale as Δρ_*xy*_ _,SMR_(ϕ) ∝ *M*_*x*_ *M*_*y*_ = *M* ^2^ cos ϕ sin ϕ,^16,17^ where ϕ denotes the angle between **M** and the current *I*. Meanwhile, the magnetization of YIG is modulated by the creation/annihilation of magnons according to *M*(*I*) = *M*_s_ + Δ*M*(*I*) sin ϕ, where *M*_s_ is the static magnetization and Δ*M*(*I*) the amplitude of the modulation induced by *I*. sin ϕ describes the angular dependence of the spin-flip efficiency, which is maxima when **M** is collinear to the spin accumulation in Pt, i.e., for ϕ = 90 and 270°. Note that the sign of Δ*M*(*I*) sin ϕ reflects whether magnons are created (negative) or annihilated (positive), which inverts when the current polarity changes or ϕ rotates by 180°. To leading order, the modulation of SMR due to magnon creation/annihilation reads as

$$\Delta \rho_{xy,\mathrm{SMR}}\left(\phi \right)\propto {M_{s}}^{2}\;\cos\phi \;\sin\;\phi +2M_{s}\Delta M\left(I\right)\;\cos\phi \;\sin^{2}\phi =R_{xy,\mathrm{SMR}}^{1\omega }\;\cos\phi \;\sin\phi +R_{xy,\mathrm{SMR}}^{2\omega }\;\cos\phi \;\sin^{2}\phi$$ 1

where *R*_*xy*,SMR_^1ω^ is the usual SMR, whereas *R*_*xy*,SMR_^2ω^ is the nonlinear term associated with magnon creation/annihilation processes and manifests in the second harmonic. As we will see below, this term is linear with *I* but becomes nonlinear when approaching the damping compensation point. In YIG/Pt, the amplitude of the magnetization variation can be evaluated by

2 $$\frac{\Delta M}{M_{s}}=R_{xy,\mathrm{SMR}}^{2\omega }/2R_{xy,\mathrm{SMR}}^{1\omega }$$

In YIG/Pt, the second-harmonic Hall response *R*_*xy*_^2ω^(ϕ) includes field-like (FL) spin–orbit torque *R*_FL_^2ω^ and spin Seebeck effect (SSE) *R*_SSE_^2ω^ contributions, whereas the antidamping torque is negligible due to its weak anomalous Hall effect.^13^ Including the nonlinear SMR (second term in eq 1), the angular dependence of *R*_*xy*_^2ω^ in YIG/Pt reads as

3 $$R_{xy}^{2\omega }\left(\phi \right)=\left(R_{xy,\mathrm{SMR}}^{2\omega }-R_{xy,\mathrm{FL}}^{2\omega }+R_{xy,\mathrm{SSE}}^{2\omega }\right)\;\cos\phi +\left(2R_{xy,\mathrm{FL}}^{2\omega }-R_{xy,\mathrm{SMR}}^{2\omega }\right)\;\cos^{3}\phi$$

## Modeling Δ*M*/*M*_s_ from Spin-Flip Scattering at the YIG/Pt Interface

Δ*M* is directly proportional to changes in the magnon spectral density *n*. The latter is modified by spin-flip scattering processes according to^18,19^ $\frac{\partial n}{\partial t}=\frac{\partial n}{\partial t_{\mathrm{rel}}}+\frac{\partial n}{\partial t_{\mathrm{sc}}}$ , where $\frac{\partial n}{\partial t_{\mathrm{rel}}}=-\frac{n-n_{0}}{\tau }$ is the magnon relaxation term in the relaxation time approximation and $\frac{\partial n}{\partial t_{\mathrm{sc}}}=\varepsilon \left(\mu_{s}-\mu_{m}\right)n$ is the effect of the interfacial spin current. *n*_0_ is the magnon spectral density at equilibrium, τ is the relaxation time, μ_s_ and μ_m_ are the current-driven nonequilibrium spin accumulation and magnon chemical potential at the Pt and YIG sides, respectively, and ε is a proportionality factor that depends on the real part of the spin mixing conductance *g*_r_ and the magnetization and thickness *t* of the magnetic film, following ε ∝ *g*_r_/*M*_s_ *t*.^20^ Both μ_s_ and μ_m_, to leading order and for moderate magnon excitations, are linear with the current. Hence, the chemical potential difference at the interface is described by μ_s_ – μ_m_ = β*I*, with β a factor that depends on Pt.

The stationary solution is $n\left(\omega ,I\right)=\frac{n_{0}}{1-I/I_{c}}$ , where *I*_c_ = 1/εβτ is the critical current for damping compensation. The variation of the magnon spectral density Δ*n* by an alternating current (ac) is given by 2Δ*n* = *n*(ω,*I*) – *n*(ω,–*I*) = 2*n*_0_ $\frac{I/I_{c}}{1-{\left(I/I_{c}\right)}^{2}}$ . By considering τ ∝ α^–1^, with α the Gilbert damping,^19^ μ_s_ ∝ *I*, and that *n*_0_ relates to the interface spin conductance *g*_s_ ∝ *g*_r_ as *n*_0_ ∝ *g*_s_ *M*_s_/*g*_r_, we find that^4,20^

4 $$\frac{\Delta M}{M_{s}}\propto \frac{j_{s}^{\mathrm{int}}}{\mu_{s}t\alpha M_{s}}\frac{I}{\left[1-{\left(I/I_{c}\right)}^{2}\right]}$$

where *j*_s_^int^ = *g*_s_(μ_s_ – μ_m_) is the spin current across the YIG/Pt interface.

## Experiment

Harmonic Hall measurements in YIG/Pt were performed in Hall bar-shaped devices (Figure 1a) consisting of epitaxial YIG films of different thicknesses interfaced with a 4-nm-thick Pt overlayer (see Methods). The magnetic, crystallographic, topographic, and interface properties of the films were investigated, and the films were found to have state-of-the-art characteristics^21,22^ (Sections S1–S4). The static, dynamic, and thermoelectric responses of the films to an ac current are extracted from first- and second-harmonic hall voltage analysis, including the nonlinear magnetoresistance originated from magnon creation/annihilation [eqs 1–4] (see Methods). For simplicity, here we focus on the Hall response *R*_*xy*_^2ω^(ϕ), although magnon effects also exhibit a longitudinal counterpart.^13,23^

**Figure 1:**
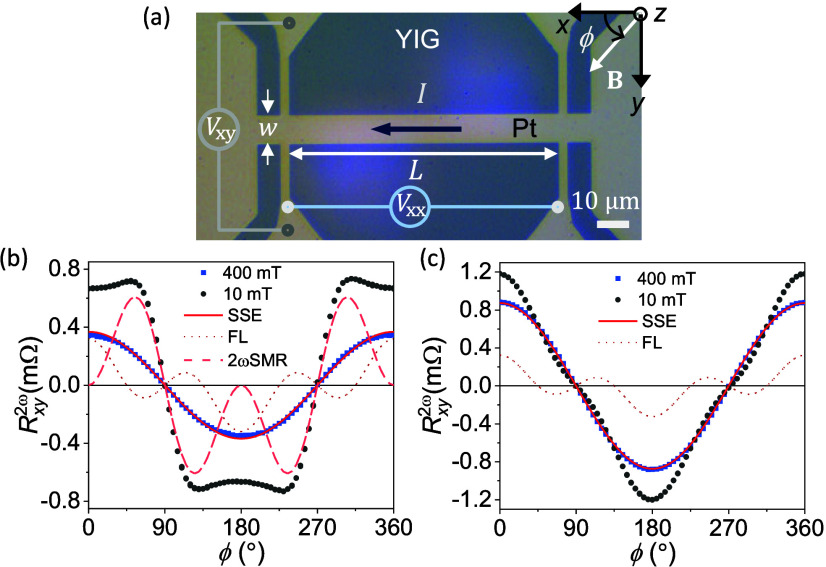
(a) Optical image of a Hall bar device (*w* = 10 μm and *L* = 100 μm) showing the coordinate system, rotation angle, and wiring. *R*_*xy*_^2ω^ is obtained from the 2ω voltage response as *V*_*xy*_^2ω^/*I*. (b and c) *R*_*xy*_^2ω^(ϕ) for YIG (10 nm)/Pt and YIG (100 nm)/Pt at 10 mT (black dots) and 400 mT (blue squares), respectively, for *I* = 4 mA. The FL, 2ωSMR, and SSE (dotted, dashed, and solid lines, respectively) represent the *R*_*xy*,FL_^2ω^, *R*_*xy*,SMR_^2ω^, and *R*_*xy*,SSE_^2ω^ contributions to *R*_*xy*_^2ω^ at 10 mT evaluated from fitting the data to eq 3. The SSE contribution overlaps with *R*_*xy*_^2ω^(400 mT), showing that at large fields the signal is dominated by the SSE.

## Results and Discussion

We find that magnon creation/annihilation effects are strongly dependent on the magnetic field and YIG thickness. Figure 1b,c show that at large fields both *R*_*xy*,FL_^2ω^ and *R*_*xy*,SMR_^2ω^ become negligible, only prevailing the spin thermoelectric contribution *R*_*xy*,SSE_^2ω^ (red line). The suppression of *R*_*xy*,FL_^2ω^ with the field agrees with the 1/*B* dependence of the torques^24^ (blue triangles, Figure 2a), but the suppression of *R*_*xy*,SMR_^2ω^ (Figure 1b) is surprising because no modulation is expected for thermal magnons. That is because the Zeeman energy μ_0_ *HM* for a magnetic field μ_0_ *HM* = 400 mT is orders of magnitude smaller than the thermal energy *k*_B_ *T*, resulting in a negligible modulation of the thermal magnon modes. In contrast, for magnon energies in the range of μ_0_ *HM*, noticeable changes are expected with the field. Therefore, the suppression of *R*_*xy*,SMR_^2ω^(*B*) indicates that the dominant magnon modes responsible for interface spin-flip scattering processes are deep subthermal, i.e., magnons in the gigahertz regime with an associated temperature *T* ^eff^ in the Kelvin regime.

**Figure 2:**
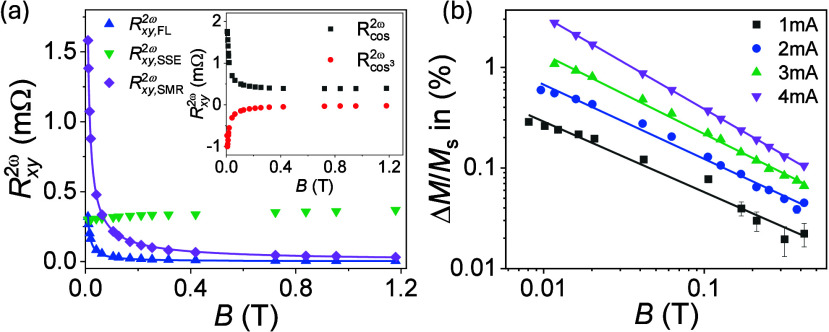
(a) Field dependence of the amplitude of the different contributions to *R*_*xy*_^2ω^(ϕ) in YIG (10 nm)/Pt. Blue and purple lines show the fit to *B* ^–γ^ with γ = 1.00 and 0.83, respectively. Inset: Amplitude of the first (black dots) and second (red dots) terms in eq 3. (b) Δ*M*/*M*_s_ vs *B* in YIG (10 nm)/Pt at different currents computed from *R*_*xy*,SMR_^2ω^ using eq 2. The solid lines show the fit to *B* ^–γ^.

In thin films and for small magnetic fields, *R*_*xy*_^2ω^ is pronounced and dominates *R*_*xy*,SMR_^2ω^(ϕ) (Figures 1b and 2a). In contrast, in 100-nm-thick YIG, *R*_*xy*,SMR_^2ω^ is negligibly small even for small magnetic fields (Figure 1c), recovering the bulk-like behavior for *R*_*xy*_^2ω^ dominated by *R*_*xy*,SSE_^2ω^ and *R*_*xy*,FL_^2ω^ in YIG/Pt.^25^ The strong suppression of *R*_*xy*,SMR_^2ω^ in 100-nm YIG (∼2 orders of magnitude compared to 10 nm) indicates that the efficiency to modulate nonequilibrium magnons by spin currents is strongly influenced by additional effects beyond thickness changes (eq 4). We will address this question in what follows.

An important step to analyze $\frac{\Delta M}{M_{s}}$ is to identify *R*_*xy*,SMR_^2ω^ from *R*_*xy*_^2ω^(ϕ). This is done by performing *R*_*xy*_^2ω^(ϕ) measurements at different magnetic fields. Because *R*_*xy*,FL_^2ω^/*R*_*xy*,SMR_^1ω^ ∝ 1/*B* ^24^ and *R*_*xy*,SSE_^2ω^(*B*) exhibits a weak field dependence, we first estimate *R*_*xy*,FL_^2ω^(*B*) by fitting the cos ϕ + cos^3^ ϕ terms in *R*_*xy*_^2ω^(ϕ), normalized by *R*_*xy*,SMR_^1ω^ [i.e., (*R*_*xy*,SSE_^2ω^ + *R*_*xy*,FL_^2ω^)/*R*_*xy*,SMR_^1ω^; eq 3], to a 1/*B* function for *B* → 0. This allows us to disentangle the different contributions to *R*_*xy*_^2ω^(ϕ) and extract *R*_*xy*,SMR_^2ω^(*B*) and *R*_*xy*,SSE_^2ω^(*B*) without requiring saturation values at large fields. See Section S5 for a detailed description of the fitting procedure along with additional *R*_*xy*_^2ω^(ϕ) data. The results for YIG (10 nm)/Pt are presented in Figure 2a.

The analysis of *R*_*xy*,FL_^2ω^ confirms that the FL torque in YIG/Pt is dominated by the Oersted field *B*_Oe_.^25^ In 10-nm YIG, we obtain *B*_FL_ + *B*_Oe_ = 0.224 ± 0.003 mT for *I* = 4 mA (blue line Figure 2a), which is consistent with $B_{\mathrm{Oe}}=\frac{I\mu_{0}}{2w}=0.25\pm 0.03$ mT. Similar *B*_FL_ + *B*_Oe_ values were obtained in all samples, corroborating the negligible interface contribution to torques, *B*_FL_, in YIG/Pt (Table S3).

*R*_*xy*,SSE_^2ω^(*B*) (green dots, Figure 2a) evolves with the field as *R*_*xy*,SMR_^1ω^(*B*), indicating that both magnitudes correlate with *M*(*B*) (Section S6). This shows that the SSE in thin films is approximately constant once the magnetization is saturated, consistent with previous results.^26^ As expected, we also found that the SSE amplitude increases linearly with the current and decreases with the YIG thickness (Figure S5). Finally, we found that *R*_*xy*,SMR_^2ω^ follows a power-law dependence on the magnetic field, *B* ^*–γ*^, with γ ranging from 0.70 at 1 mA to 0.83 at 4 mA for 10-nm YIG (Figure 2a,b). This analysis shows that the different contributions to *R*_*xy*_^2ω^ can be disentangled, allowing for quantification of the magnon creation/annihilation processes. Furthermore, the nonlinear SMR was also evaluated from *R*_*xy*_^2ω^(ϕ), yielding consistent results (Section S7).

The variation of the magnetization Δ*M*/*M*_s_ in thin films exhibits a nonlinear response with the current and varies with the magnetic field (Figure 3). The current dependence is consistent with eq 4, which is valid when the channel width *w* is much larger than the magnon diffusion length λ_m_. This scenario applies to our samples because λ_m_ ∼ 1.3 μm for 100-nm YIG and gradually decreases in thinner films,^10^ making it possible to reach damping compensation in a continuous magnetic film.^27^

**Figure 3:**
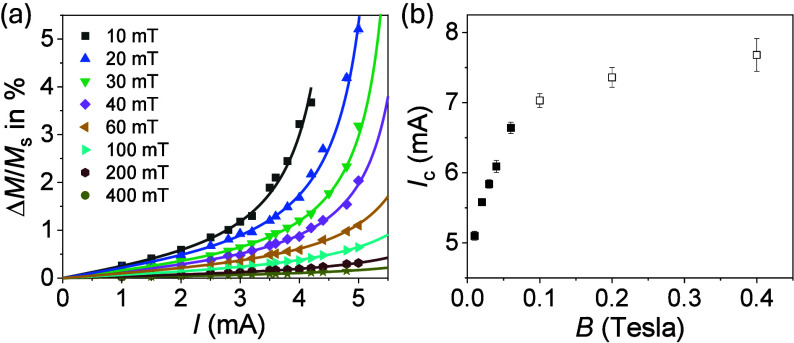
(a) Current dependence of Δ*M*/*M*_s_ in YIG (10 nm)/Pt at different magnetic fields. Solid lines show the fits to eq 4. (b) Field dependence of *I*_c_ extracted from panel a. Open dots show estimates of *I*_c_ for magnetic fields where Δ*M*/*M*_s_ does not show evident nonlinear behavior. Error bars represent the uncertainty associated with the fits.

The critical current for damping compensation increases as the magnetic field increases (Figure 3b). Up to ∼40 mT, *I*_c_ can be reliably estimated, following a linear increase with the field. At larger fields, however, $\frac{\Delta M}{M_{s}}\left(I\right)$ becomes flatter (Figure 3a), making the estimation of *I*_c_ inaccurate (open dots, Figure 3b). The strong field dependence of *I*_c_ supports the idea that the magnons contributing to Δ*M* must be deep subthermal. Indeed, the increase of *I*_c_ with the field is consistent with the expected increase of Gilbert damping for low-frequency magnons.^28^ The absence of an upturn of *I*_c_ at small fields indicates that crystal inhomogeneities are remarkably small,^27^ in agreement with X-ray diffraction (XRD) measurements (Section S2). The dominant role of subthermal magnons to long-range diffusive transport, current-induced condensation effects, and SSE phenomena are well-known.^26,29–35^ Our results indicate that subthermal magnons also play a key role in determining the spin-flip scattering efficiency at interfaces.

Δ*M* exhibits an exponential dependence on magnetic field *B* ^–γ^, with γ strongly influenced by the YIG thickness and current (Figure 4). The suppression is large in thin films and increases with the current, reaching γ values ∼1 in 10-nm YIG. In contrast, for small drives and thick layers, γ drops to ∼0.1. A similar exponential behavior has also been observed for the unidirectional SMR originated from spin-flip scattering processes,^36^ with the exponent following the same trend with *I* and *t* as we observe for γ­(*I*,*t*) (Figure 4c). The results were attributed to changes in the magnon stiffness *D*, as is expected to decrease with reducing thickness or increasing current. *D* can affect Δ*M* through τ­(*B*), yielding Δ*M* ∝ 1/(1 + ξ*B*) for low-frequency magnons, with ξ a parameter that depends on *D* (Section S8). However, such a field dependence produces poor fits to the experimental data, indicating that *D* is not the dominant parameter that controls Δ*M*(*B*).

**Figure 4:**
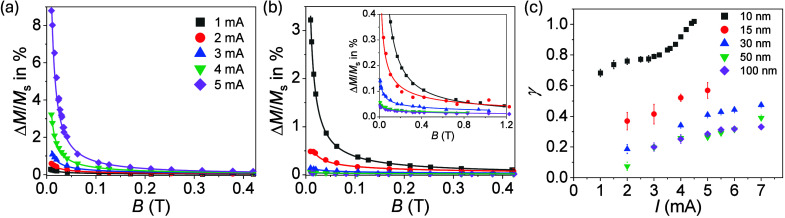
(a and b) Magnetic field dependence of Δ*M*/*M*_s_ evaluated from *R*_*xy*_^2ω^(ϕ) for different currents (*t* = 10 nm) and YIG thicknesses (*I* = 4 mA). The solid lines are fits to *B* ^–γ^. Inset in panel b: Zoom in the small Δ*M*/*M*_s_ amplitude range. Data for 15, 30, 50, and 100 nm are scaled to account for changes in the interface quality relative to 10-nm YIG (Section S4). (c) γ values extracted from data fits at different currents and thicknesses.

The exponential dependence of Δ*M*(*B*) can be explained by the field dependence of the spin conductance *g*_s_(*B*). Because $\frac{\Delta M}{M_{s}}\propto j_{s}^{\mathrm{int}}=g_{s}\left(\mu_{s}-\mu_{m}\right)$ (eq 4), and μ_s_ – μ_m_ should remain constant or increase with *B* (at leading order, only μ_m_ is modulated with field, with an expected decrease correlated with $\frac{\Delta M}{M_{s}}$ ), the suppression of *g*_s_ must be the dominant mechanism behind the observed $\frac{\Delta M}{M_{s}}\left(B\right)$ (Figure 4). At *k* = 0, *g*_s_ is connected to the magnetization via *g*_s_ ∝ ⟨*M*_⊥_^2^⟩,^15^ i.e., the average of the quadratic magnetic components perpendicular to the magnetization. In essence, this term captures the degree of magnetic disorder (magnon occupation). For instance, ⟨*M*_⊥_^2^⟩, and thus *g*_s_, increases with the current (i.e., temperature), as observed in experiments^30,37^ (Figure 4a,c and Section S9). Interestingly, ⟨*M*_⊥_^2^⟩(*B*) can be qualitatively estimated from *R*_*xy*,SMR_^1ω^(*B*). Considering *R*_*xy*,SMR_^1ω^(*B*) = *R*_*xy*,SMR_^1ω,sat^ – Δ*R*_*xy*,SMR_^1ω^(*B*), with *R*_*xy*,SMR_^1ω,sat^ denoting the asymptotic resistivity value at large fields, the second term captures the degree of disorder with Δ*R*_*xy*,SMR_^1ω^(*B*) ∝ ⟨*M*_⊥_^2^⟩(*B*) ∝ *g*_s_(*B*). The analysis reveals an exponential decay of Δ*R*_*xy*,SMR_^1ω^(*B*) of the form *B* ^–η^, with η values exhibiting a consistent trend with the thickness and current, as observed for γ (Figure S8 and Tables S4 and S5). This suggests that the suppression of Δ*M* is dominated by modulation of the interface spin conductance with the field, further highlighting the key role of subthermal magnons in spin-flip scattering processes. We note that τ­(*B*) and μ_m_(*B*) may also contribute to Δ*M*(*B*)/*M*_s_ (eq 4), which can explain the quantitative discrepancy between γ and η. The demonstrated modulation of *g*_s_ with *B* is also expected to influence other spin-driven magnon effects, such as the amplitude of magnon signals in nonlocal devices due to their *g*_s_^2^ dependence. This effect is expected to be significant (negligible) for thin (thick) magnetic layers and large (small) drives, where large (small) γ factors are expected (Figure 4c), in agreement with the observations.^11,30,31^

Finally, we analyze the role of the YIG thickness on the amplitude of Δ*M*/*M*_s_. Figure 5a shows the current dependence of Δ*M*/*M*_s_ at 10 mT, where a very strong thickness dependence is inferred. Notably, in contrast to the 10-nm sample, *I*_c_ cannot be experimentally reached for 15-nm YIG. Moreover, for *t* ≥ 30 nm, Δ*M*/*M*_s_ is orders of magnitude smaller than that for 10-nm YIG and exhibits a nearly linear dependence with *I*, indicating that $\frac{I}{I_{c}}\ll 1$ . A current dependence and an amplitude for Δ*M*/*M*_s_ similar to those of our YIG (10 nm)/Pt were also observed in PLD-grown YIG (6.2 nm)/Pt.^13^ Using eq 4, in the linear regime ( $\frac{I}{I_{c}}\ll 1$ ),

5 $$\frac{\Delta M_{1}/M_{s1}}{\Delta M_{2}/M_{s2}}=\frac{\alpha_{2}t_{2}}{\alpha_{1}t_{1}}\;\frac{M_{s2}}{M_{s1}}\;\frac{j_{s1}^{\mathrm{int}}}{j_{s2}^{\mathrm{int}}}\approx \frac{M_{s2}}{M_{s1}}\;\frac{j_{s1}^{\mathrm{int}}}{j_{s2}^{\mathrm{int}}}$$

where the numbers denote the samples and $\frac{\alpha_{2}t_{2}}{\alpha_{1}t_{1}}∼1$ due to the dominant 1/*t* dependence of α for the thicknesses explored^38^ (Section S10). Therefore, the large differences observed in Figure 5a are essentially associated with the difference in the interface spin current $\frac{j_{s1}^{\mathrm{int}}}{j_{s2}^{\mathrm{int}}}$ modulated by $\frac{M_{s2}}{M_{s1}}$ and the nonlinearity of Δ*M*(*I*) as *I* → *I*_c_. The latter is estimated from fitting of the data to eq 4 (solid lines Figure 5a).

**Figure 5:**
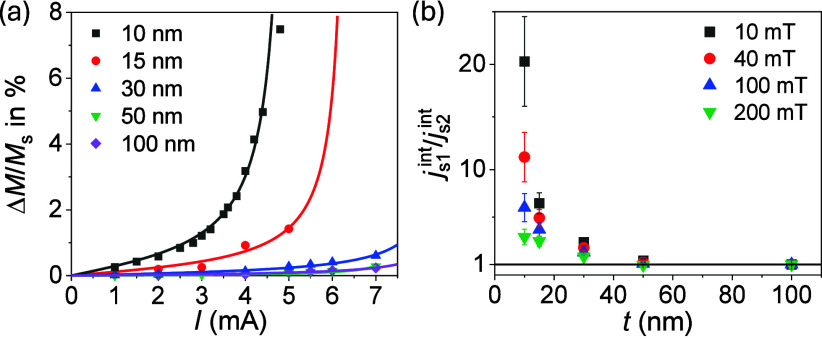
Current dependence of Δ*M*/*M*_s_ for different YIG thicknesses. The amplitudes for *t* = 15, 30, 50, and 100 nm are scaled according to variations in the interface quality relative to 10-nm YIG, which is evaluated from *R*_*xy*,SMR_^1ω^ (Section S4). Solid lines present the fits to eq 4. (b) Estimates of *j*_s1_^int^/*j*_s2_^int^ (eq 5), taking 100-nm YIG as in ref 2.

Figure 5b presents the enhancement of the interface spin current when reducing the YIG thickness. In bulk materials, $\frac{j_{s1}^{\mathrm{int}}}{j_{s2}^{\mathrm{int}}}∼1$ , indicating that the enhancement of *j*_s_^int^ arises from vertical confinement. This cannot be attributed to the reduction of *T*_c_ in films. Assuming a ${\left(T/T_{c}\right)}^{3/2}$ dependence on *g*_s_,^4^ we expect $\frac{g_{s1}}{g_{s2}}∼1.58$ for 10-nm YIG compared to 100 nm, which is about a factor of 13 smaller than $\frac{j_{s1}^{\mathrm{int}}}{j_{s2}^{\mathrm{int}}}$ at 10 mT (Figure 5b and Section S11). Moreover, increasing *B* leads to the reduction of $\frac{j_{s1}^{\mathrm{int}}}{j_{s2}^{\mathrm{int}}}\to 1$ , indicating that the suppression of low-frequency magnons results in the homogenization of the response across all thicknesses as expected.

Both the discretization of the magnon bands and changes in μ_s_ – μ_m_ with *t* may contribute to *j*_s_^int^(*t*). Because $\frac{\Delta M}{M_{s}}$ is increasing as the YIG thickness decreases (Figure 5a), μ_s_ – μ_m_ must decrease, resulting in $\frac{g_{s1}}{g_{s2}}≳\frac{j_{s1}^{\mathrm{int}}}{j_{s2}^{\mathrm{int}}}$ . This highlights the dominant role of *g*_s_(*t*) on the interfacial spin current transmission. Our findings demonstrate that the spin transmission across interfaces is enhanced with decreasing film thickness, thereby enhancing magnon transport signals and facilitating damping compensation.

For subthermal magnons with energy *k*_B_ *T* ^eff^, the number of occupied magnon bands is given by^11^ $N=1+\mathrm{int}\left(\frac{t}{\pi }\sqrt{\frac{k_{B}T^{\mathrm{eff}}}{\hbar \gamma_{m}D}}\right)$ , where γ_m_ is the gyromagnetic ratio and *D* = 5 × 10^–17^ Tm^2^ for YIG.^39^ We speculate that the increase of *j*_s_^int^(*t*) upon reducing the thickness (Figure 5b) is due to the vertical confinement of the subthermal magnon modes, leading to the occupation of only a few or a single magnon band (Section S12). In fact, the 3D → 2D transition is expected to occur at a length scale $\lambda_{T^{\mathrm{eff}}}=\sqrt{\frac{4\pi \hbar \gamma_{m}D}{k_{B}T^{\mathrm{eff}}}}$ . For instance, for magnons with *T* ^eff^ ∼ 10 K (1 K), λ_Teff_ is ∼10 nm (100 nm), suggesting that the observed steep increase of $\frac{j_{s1}^{\mathrm{int}}}{j_{s2}^{\mathrm{int}}}$ in thin films (Figure 5b) is due to the dominant role of deep subthermal magnons on *g*_s_.

The key role of low-frequency magnons on the amplitude of the SSE is well-established.^26,34,35^ However, modulation of the interfacial spin transmission efficiency reported here exhibits clear differences from the SSE. First, the SSE is driven by a temperature gradient and involves magnons across the full spectrum,^40,41^ while spin-flip scattering is driven by the spin Hall effect (SHE), having different associated energies. As a result, we find that *j*_s_^int^(*B*) ∝ *g*_s_(*B*) due to modulation of the low-frequency magnons, leading to a strong suppression of Δ*M*/*M*_s_ with the field. In contrast, the SSE remains nearly field independent, consistent with its thermal origin. Second, the SSE decreases with reduced YIG thickness due to the suppression of low-frequency magnons^26,34^ (Figure S5a). This behavior is in striking contrast to the observed enhancement of magnon creation/annihilation processes upon a reduction in the YIG thickness, highlighting the fundamentally different physics governing spin-flip scattering processes driven by spins or temperature.

The model proposed here is valid for ferro/ferrimagnets where *g*_s_ ≪ *g*_r_ and hence magnon excitations are weak compared to *M*_s_, leading to SMR ∝ *M*_s_^2^.^16,17^ This occurs at most experimental conditions like the ones studied here, but in the vicinity of phase transitions or compensation points, strong nonlinear magnon phenomena emerge,^20^ leading to enhanced scattering processes or the loss of the collinear order in ferrimagnets. These scenarios are not captured by our model and deserve a dedicated independent study. In addition, the methodology presented here, if adapted to materials with negligible or absent net magnetic moment such as antiferromagnets, could be used to investigate magnon excitations in emerging materials such as canted antiferromagnets and altermagnets.

## Conclusions

We analyzed changes in the magnetization driven by spin-flip scattering at YIG/Pt interfaces and found that the amplitude of the effect is dramatically enhanced in thin films. The phenomenon is explained via the strong thickness and field dependence of the interfacial spin-current transmission efficiency, which to date has been considered to depend on thermal magnons for energies larger than the Zeeman energy.^4,11^ We attribute this finding to the unexpected dominant role of deep subthermal magnons in spin-flip scattering processes. Our work shows that magnon transport, auto-oscillation, and magnon condensation effects driven by the SHE can be dramatically enhanced by geometrical confinement.

## Methods

### Samples Preparation

Epitaxial YIG films of thicknesses *t* = 100, 50, 30, 15, and 10 nm were grown via sputtering deposition onto (111)-oriented Gd_3_Ga_5_O_12_ substrates from a stochiometric Y_3_Fe_5_O_12_ target in a system with a base pressure of <1 × 10^–5^ Pa. The substrate temperature was maintained at 800 °C and the Ar pressure to 1.3 Pa during growth. After deposition, the samples were annealed for 30 min at the deposition temperature and finally cooled back to room temperature in vacuum. A 4-nm-thick polycrystalline Pt layer was subsequently sputtered at room temperature at 0.4 Pa Ar atmosphere. Pt Hall bars were patterned using optical lithography and Ar plasma etching, leaving the YIG films unetched.

### Experiment

Harmonic transport measurements were performed in a magnetotransport setup capable of applying a 2T magnetic field and rotating the sample by 360°. An ac current *I* of frequency ω = 10 Hz was applied, and both the longitudinal and transverse responses were captured in a 205 kHz acquisition card. The harmonic components to the transport were obtained by demodulating the voltage at frequency ω. In all measurements, the magnetic field applied was much larger than the in-plane anisotropy field of YIG,^21,22^ resulting in a collinear alignment of **M** with **B**. Therefore, ϕ in eqs 1 and 2 is directly determined by **B** (Figure 1a).

### Error Analysis

The error bars associated with the data points in Figures 2–5 are displayed or are smaller than the size of the dots. Error bars are calculated from the uncertainty of the angular-, current-, and magnetic field-dependent fits of the linear and nonlinear magnetoresistance measurements to eqs 1–5 and error propagation.

## Supplementary Material



## Data Availability

The data that support the findings of this work are available at https://doi.org/10.21950/1RVPMU.

## Notes

SQUID magnetometry, XRD and AFM characterization of the films, analysis of the YIG/Pt interface quality *g*_r_, details on the analysis of *R*_*xy*_^1ω^(ϕ) and *R*_*xy*_^2ω^(ϕ) and additional magnetoresistance data, analysis and discussion of *R*_*xy*,FL_^2ω^(ϕ) and *R*_*xy*,SSE_^2ω^(ϕ), analysis of *R*_*xx*,SMR_^2ω^(ϕ), discussion on the role of stiffness on Δ*M*(*B*), field dependence of *R*_*xy*_^1ω^(ϕ), Gilbert damping of YIG­(*t*)/Pt films, characterization of Joule heating and discussion on *j*_s_^int^, magnon band occupation with *t* and *T* ^eff^, and refs 42–54 (PDF)
