# Supplementary material for: Large Enhancement of Spin-Flip Scattering Efficiency at Y3Fe5O12/Pt Interfaces Due to Vertical Confinement
Source: Nano Lett. 2026 May 13;26(20):6525–31. doi: 10.1021/acs.nanolett.5c05598 (PMC13220310; doi:10.1021/acs.nanolett.5c05598)
Supplement: Supplementary file 1 [file nl5c05598_si_001.pdf]

Supporting Information

Large enhancement of spin-flip scattering efficiency at $Y_3Fe_5O_{12}$/Pt interfaces due to vertical confinement

Haripriya Madathil[1], Pranav Pradeep[1], Paul Noël[2], and Saül Vélez[1,3,*]

[1] Spintronics and Nanodevices Laboratory, Departamento de Física de la Materia Condensada and Condensed Matter Physics Center (IFIMAC), Universidad Autónoma de Madrid, E-28049 Madrid, Spain

[2] Université de Strasbourg, CNRS, IPCMS UMR 7504, Strasbourg F-67034, France

[3] Instituto Nicolás Cabrera, Universidad Autónoma de Madrid, E-28049 Madrid, Spain

*saul.velez@uam.es

TABLE OF CONTENTS:

S1. Magnetic characterization of the YIG films

S2. X-ray diffraction measurements

S3. Topographic characterization

S4. Evaluation of g_r

S5. Additional $R_{xy}^{2\omega}$ data and description of analysis procedure

S6. Analysis of $R_{xy,SSE}^{2\omega}$

S7. Non-linear SMR from longitudinal $R_{xx}^{2\omega}$ measurements

S8. The role of stiffness in the field dependence of ΔM

S9. Field dependence of $\Delta R_{\mathrm{xy,SMR}}^{1\omega}$

S10. Estimation of damping values of our YIG films

S11. Joule heating and its effect on $\Delta M/M_\mathrm{s}$ and $j_\mathrm{s1}^\mathrm{int}/j_\mathrm{s2}^\mathrm{int}$

S12. The highest occupied magnon band for different t and T^eff

S1. Magnetic characterization of the YIG films

The magnetic properties of the YIG thin films were investigated via SQUID magnetometry. Fig. S1a presents the magnetic hysteresis loops as a function of magnetic field $m(H)$ taken in all the YIG patterned samples investigated in this work at 300 K. The linear paramagnetic response of the GGG substrate is subtracted for clarity. The estimated saturation magnetization M_s of the YIG films, calculated by considering the surface area of the films 5x5 mm^2, is lower than the bulk value 140 kA/m for YIG. The reduction of M_s in thin films due to finite size effects and the formation of dead layers is well known[1–3]. For instance, the interdiffusion of Gd and Y at the GGG/YIG interface, as well as the exposure of the YIG surface area surrounding the devices to the Ar plasma for etching the Pt may result in the formation of a magnetic dead layer having a softer (paramagnetic-like) response[2,4,5]. Based on this, a dead layer of thickness 5 ± 1 nm was considered for all YIG samples to estimate M_s. Fig. S1b shows the M_s values at 300 K estimated from the $m(H)$ loops presented in Fig. S1a.

Hysteresis loops at different temperatures were also performed for the 10 nm YIG film. The extracted M_s(T) values are presented in Fig. S1c. We fitted the data points using the mean-field theory, which predicts following temperature dependence[6]

$$M_s(T) = M_s(0)\left(1 - (T/T_c)\right)^{1/2} \quad . \qquad \text{(S1)}$$

The data fit yields $T_c = 354 \pm 7$ and M_s(0) = 203.9±3.5 kA/m (Fig. S1c), which is consistent with the YIG bulk value for M_s(0) and a reduced T_c. The suppression of M_s and T_c upon reducing the film thickness has been observed in diverse systems, including YIG films[2,3,7]. The variation of M_s with thickness (Fig. S1b) is taken into account for the analysis of $j_{s1}^{\text{int}}/j_{s2}^{\text{int}}$ [Eq. (5) of main text], see Section S11.

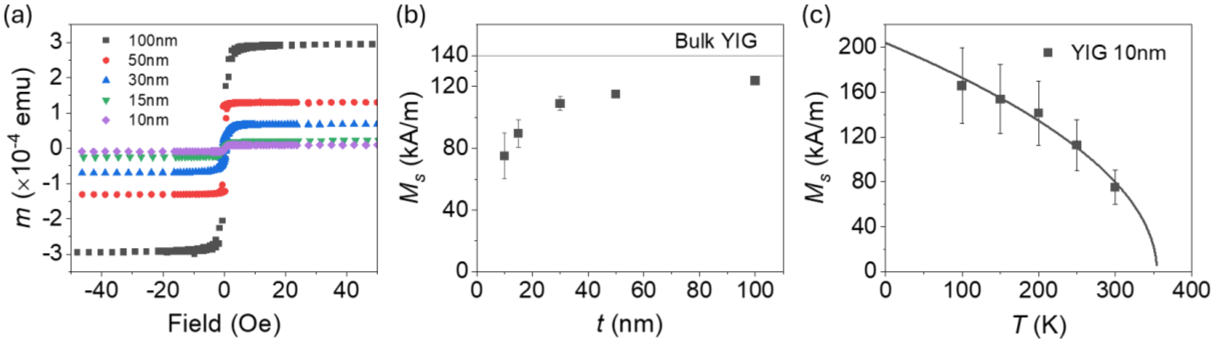

Figure S1. (a) SQUID measurements of the YIG samples measured at 300 K after subtracting the paramagnetic response of the GGG substrate. b) Thickness dependence of M_s considering a constant dead layer of 5 ± 1 nm for all the films. c) Variation of saturation magnetization M_s with temperature for YIG(10nm) measured using SQUID magnetometer. The solid line shows the fit to the Eq. (S1). The error bars in (b) and (c) account for an uncertainty of ± 1 nm in the effective thickness of the YIG layer.

S2. X-ray diffraction data

X-ray diffraction (XRD) measurements were performed at $\theta - 2\theta$ configuration. Fig. S2 shows X-ray reflectivity data obtained in a sister GGG/YIG(30nm) sample as the one explored in the main text. The scan reveals the (444) peak at $(51.08 \pm 0.02)°$ consistent with the lattice parameter of the GGG substrate, and a peak at $(50.20 \pm 0.02)°$ and corresponding Laue oscillations associated to the YIG film indicating tensile strain[2]. The appearance of clear Laue oscillations indicates the high crystalline quality of the films.

Figure S2. XRD scan of GGG/YIG(30nm). From the Laue fringe spacing, we estimated the thickness of YIG to be 33.6 ± 1.9 nm using the equation $t = \frac{\lambda}{\Lambda \cos\theta}$, where Λ is the full width at half maximum and $\lambda = 1.54$ Å is the wavelength of the x-rays used in the measurements.

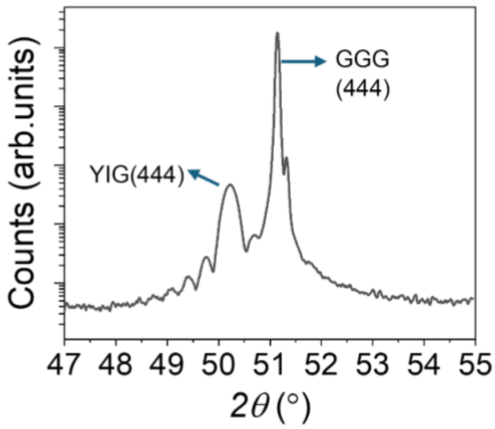

S3. Topographic characterization

The surface roughness of the films investigated in this work were characterized by atomic force microscopy (AFM). Fig. S3 shows exemplary AFM scans taken over 5 × 5 μm² areas in both the YIG (a) and Pt (b) regions. The analysis of 3 equivalent surface areas (for each case) showed that the root mean square (RMS) roughness in both YIG and Pt regions are similar and smaller than 0.5 nm in all samples, except for 15 nm YIG. In that case, the surface topography reveals the formation of small bubbles (sub-100nm in diameter), resulting in a larger RMS value on the order of 2 nm. The average values found for each case and their dispersion are summarized in Table S1.

The increased surface roughness in 15 nm YIG is consistent with the reduction of the real part of the spin mixing conductance g_r evaluated via spin Hall magnetoresistance (SMR) measurements (Section S4). We have taken into account the difference in interface quality for the analysis of the relative change in spin-flip scattering efficiency when varying the YIG thickness.

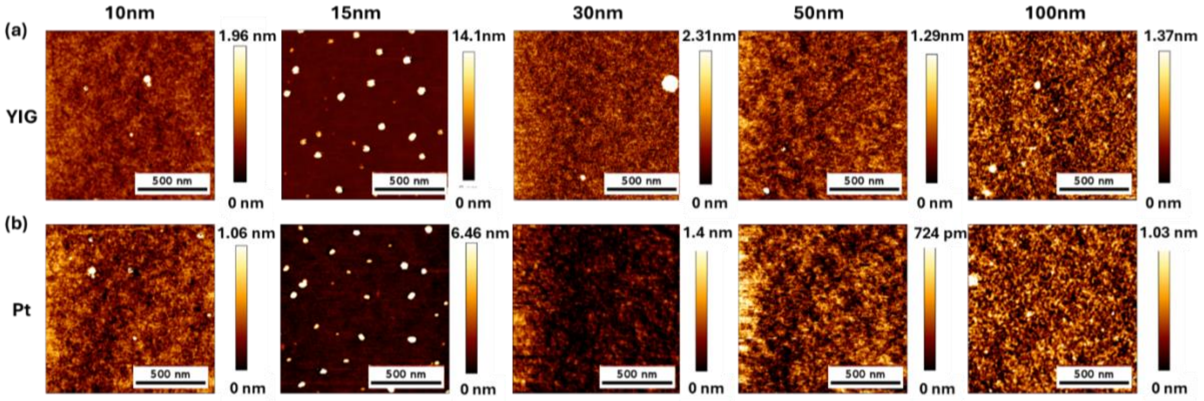

Figure S3. Topographic characterization of the YIG/Pt films investigated in this work. (a) and (b) Representative AFM images taken in YIG and Pt regions, respectively, over a surface area of 5 × 5 μm². From left to right, images taken in 10, 15, 30, 50, and 100-nm-thick YIG (top) and YIG/Pt (bottom) regions.

YIG thickness (nm)	RMS (nm) YIG	RMS (nm) Pt capped YIG
10	0.4±0.1	0.4±0.2
15	2.3±0.1	2.0±1.1
30	0.5±0.1	0.2±0.1
50	0.4±0.2	0.3±0.2
100	0.3±0.1	0.4±0.1

Table S1. Summary of the RMS roughness values obtained on YIG and Pt-capped surface areas for all the films studied in this work. The error bars are calculated from the standard deviation of the RMS values obtained in 3 equivalent regions for each case.

S4. Evaluation of g_{r}

The YIG/Pt interface quality can be characterized by SMR[8,9]. To leading order, the amplitude of the SMR is proportional to the spin mixing conductance g_{r}, which parameterizes the spin transmission efficiency at the interface. Therefore, by comparing the amplitude of $R^{1\omega}_{xy,SMR}$ between the samples, we can characterize the relative change in g_{r}. Accordingly, we calculated $g^*_{\mathrm{r}} = \frac{R^{1\omega}_{xy}(\mathrm{t})/R_{\mathrm{L}}(t)}{R^{1\omega}_{xy}(\mathrm{t}=10)/R_{\mathrm{L}}(t=10)}$, which parametrizes the relative change in g_{r} compared to 10nm thick YIG, where R_{L} refers to the longitudinal resistance. Since g_{s} is proportional to g_{r}[10], we have scaled the $\Delta M/M_{\mathrm{s}}$ data using the factor g^*_{r} to analyze the intrinsic change in magnon creation-annihilation efficiency associated to the YIG thickness.

Table S2. Summary of the measured R_L and $R^{1\omega}_{xy}$ values in all samples investigated in this work along with the relative changes in interfacial spin conductance, g^*_r, compared to 10 nm YIG.

t (nm)	R_{L}(Ohms)	$R^{1\omega}_{xy}$ (mOhms)	g^*_{r}
10	960$\pm$1	29.4$\pm$ 0.5	1
15	1000$\pm$1	10.2$\pm$0.4	0.33$\pm$0.03
30	800$\pm$1	22.7$\pm$0.4	0.94$\pm$0.02
50	773$\pm$1	39.8$\pm$0.4	1.70$\pm$0.02
100	720$\pm$1	41.0$\pm$0.4	1.85$\pm$0.02

S5. Additional $R^{2\omega}_{xy}$ data and description of analysis procedure

Here we present additional harmonic Hall data obtained at different magnetic fields and provide a detailed description of the analysis methodology followed to extract the different components to $R^{2\omega}_{xy}(\phi)$ [Eq. (3) of the main text]. Fig. S4a and Fig. S4b show the first- $R^{1\omega}_{xy}(\phi)$ (Fig. S4a) and second-harmonic $R^{2\omega}_{xy}(\phi)$(Fig. S4b) response in 10 nm YIG measured at different magnetic fields. From $R^{1\omega}_{xy}(\phi)$ we can extract the linear SMR amplitude, whereas $R^{2\omega}_{xy}(\phi)$ in YIG/Pt has contributions from the field-like torque ($R^{2\omega}_{xy,\mathrm{FL}}$), the non-liner SMR associated to the creation and annihilation of magnons ($R^{2\omega}_{xy,\mathrm{SMR}}$), and the spin Seebeck effect ($R^{2\omega}_{xy,\mathrm{SSE}}$). See Refs.[11,12] and Section *Non-linear magnetoresistance due to magnon creation/annihilation* of the main text for additional discussion.

In Fig. S4b we observe the gradual evolution of the $R^{2\omega}_{xy}(\phi)$ signal with field as complementary to the data presented in Fig. 1b of the main text. In the small field regime, all contributions are significant, yielding to pronounced $\cos\phi$ ($R^{2\omega}_{\cos}$) and $\cos^3\phi$($R^{2\omega}_{\cos^3}$) components (inset Fig. 2a main text). Upon increasing the magnetic field, a gradual suppression of $R^{2\omega}_{xy,\mathrm{FL}}$ and $R^{2\omega}_{xy,\mathrm{SMR}}$ takes place, resulting in the suppression of the $\cos^3\phi$ term, and only the $\cos\phi$ contribution associated to $R^{2\omega}_{xy,\mathrm{SSE}}$ survives (Figs. 1b and S4b). Since $R^{2\omega}_{xy,\mathrm{FL}}$, $R^{2\omega}_{xy,\mathrm{SMR}}$, and $R^{2\omega}_{xy,\mathrm{SSE}}$ follow different field dependences, we can extract the different components to $R^{2\omega}_{xy}$ by performing harmonic measurements at different fields and executing the following steps:

1. $R^{1\omega}_{xy}(\phi)$ is fit to $R^{1\omega}_{xy,\mathrm{SMR}}\cos\phi\sin\phi$, and $R^{2\omega}_{xy}(\phi)$ to Eq. (3) of the main text to obtain the amplitude and sign of the $\cos\phi$ ($R^{2\omega}_{\cos}$) and $\cos^3\phi$($R^{2\omega}_{\cos^3}$) components. The results from the fits of $R^{2\omega}_{xy}(\phi)$ in 10 nm YIG are presented in the inset of Fig. 2a. $R^{1\omega}_{xy,\mathrm{SMR}}(B)$ is shown in Fig. S6a.
2. Since $R^{2\omega}_{\cos} = R^{2\omega}_{xy,\mathrm{SMR}} - R^{2\omega}_{xy,\mathrm{FL}} + R^{2\omega}_{xy,\mathrm{SSE}}$ and $R^{2\omega}_{\cos^3} = 2R^{2\omega}_{xy,\mathrm{FL}} - R^{2\omega}_{xy,\mathrm{SMR}}$ [Eq. (3) main text], we compute $R^{2\omega}_{\cos} + R^{2\omega}_{\cos^3} = R^{2\omega}_{xy,\mathrm{FL}} + R^{2\omega}_{\mathrm{xy,SSE}}$. This allows removing the $R^{2\omega}_{xy,\mathrm{SMR}}$ contribution.

3. From the calculated term in point 2, we can estimate $R^{2\omega}_{xy,\mathrm{FL}}$ and $R^{2\omega}_{\mathrm{xy,SSE}}$. This is possible due to the weak field dependence of $R^{2\omega}_{\mathrm{xy,SSE}}$ in films (Refs.[13–15] and Section S6), whereas $R^{2\omega}_{xy,\mathrm{FL}} = \frac{R^{1\omega}_{xy,\mathrm{SMR}}(B_{\mathrm{FL}}+B_{\mathrm{Oe}})}{2B}$[16]. Therefore, $R^{2\omega}_{xy,\mathrm{FL}}$, and thus $(B_{\mathrm{FL}} + B_{\mathrm{Oe}})$, can be obtained from the slope of $(R^{2\omega}_{\cos} + R^{2\omega}_{\cos^3})/R^{1\omega}_{xy,\mathrm{SMR}}$ vs $1/B$ in the small field regime. The obtained values for $(B_{\mathrm{FL}} + B_{\mathrm{Oe}})$ for all the samples investigated in this work are listed in Table S3 for a current of 4 mA. We also verified that $B_{\mathrm{FL}} + B_{\mathrm{Oe}}$ is linear with the current (Fig. S4c presents the data obtained for 10 nm YIG), showing the consistency of the method.
4. By using the obtained $R^{2\omega}_{xy,\mathrm{FL}}(B)$ in Step 3, we can directly evaluate $R^{2\omega}_{xy,\mathrm{SSE}}(B)$ and $R^{2\omega}_{xy,\mathrm{SMR}}(B)$ from the $R^{2\omega}_{\cos}$ and $R^{2\omega}_{\cos^3}$ terms. We find that $R^{2\omega}_{xy,\mathrm{SSE}}(B)$ correlates with $R^{1\omega}_{xy,\mathrm{SMR}}$, showing that $R^{2\omega}_{xy,\mathrm{SSE}}(B)$ is given by $M^2(B)$ as expected for films (see Section S6). This corroborates the robustness of the analysis method to evaluate magnon creation/annihilation effects driven by spin-flip scattering processes (i.e., $R^{2\omega}_{xy,\mathrm{SMR}}$) from harmonic transport measurements.

The errors in the estimates of $R^{1\omega}_{xy,\mathrm{SMR}}$ (as well as $R^{2\omega}_{xy,\mathrm{SSE}}$ and $R^{2\omega}_{xy,\mathrm{FL}}$), and hence of $\Delta M/M_{\mathrm{s}}$, were calculated from the uncertainty of the fit of the experimental data to the $R^{1\omega}_{xy}(\phi)$ and $R^{2\omega}_{xy}(\phi)$ functions (Step 1) and error propagation.

We also note that in the absence of magnon generation via spin-flip scattering, the second harmonic response $R^{2\omega}_{xy}$ should exhibit a strictly linear dependence on the applied current[16]. The excitation and annihilation of magnons modify the magnetization dynamics, introducing a nonlinear spin magnetoresistance contribution, $R^{2\omega}_{xy,\mathrm{SMR}}$. This nonlinear term affects both the $\cos\phi$ and $\cos^3\phi$ components of $R^{2\omega}_{xy}$, leading to deviations from the expected linear scaling as shown in Fig. S4d. The non-linear scaling is due to the current-induced damping compensation, which is well captured by Eq. (4) of the main text and demonstrated in Figs. 3a and 5a of the manuscript.

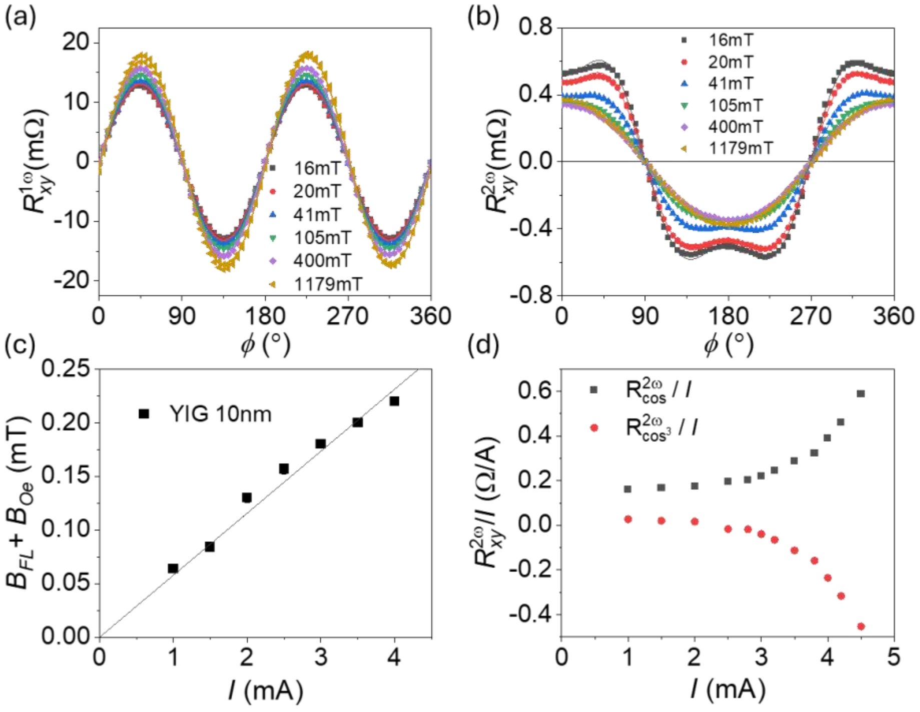

Figure S4. (a), (b) $R^{1\omega}_{xy}(\phi)$ and $R^{2\omega}_{xy}(\phi)$ in YIG(10nm)/Pt measured at different magnetic fields ranging from 16 to 1179 mT (data points). The solid lines overlapping the data points are the fits to the functions described in Step 1. (c) Current dependence of $B_{\mathrm{FL}} + B_{\mathrm{Oe}}$ extracted from $R^{2\omega}_{xy,\mathrm{FL}}$ in 10 nm YIG. The errors

associated to the data points, estimated from the uncertainty of the fit described in step 1, are smaller than the size of the dots. The straight line represents the linear fit to the data points with zero offset, from which we estimate the slope $(B_{\mathrm{FL}} + B_{\mathrm{Oe}})/I$ and associated error. Same analysis was done to all samples and the results presented in Table S3. (d) Current dependence of $R^{2\omega}_{\cos}/I$ and $R^{2\omega}_{\cos^3}/I$ in 10 nm YIG at 10 mT field. The deviation from a constant value is due to $R^{2\omega}_{xy,\mathrm{SMR}}$. The error bars associated to the uncertainty of the fits (and error propagation) in (c) and (d) are smaller than the size of the dots.

YIG thickness (nm)	$B_{\mathrm{FL}} + B_{\mathrm{Oe}}$ (mT)
10	0.224±0.003
15	0.263±0.003
30	0.207±0.002
50	0.207±0.002
100	0.187±0.003

Table S3. $B_{\mathrm{FL}} + B_{\mathrm{Oe}}$ obtained from the analysis of $R^{2\omega}_{xy}(\phi)$ at 4 mA for all the samples investigated in this work. The results show a dominant B_{Oe} contribution to $R^{2\omega}_{xy,\mathrm{FL}}$ in agreement with literature for YIG/Pt[11,17]. Here $B_{\mathrm{Oe}} = I\mu_0/2w = 0.25 \pm 0.03$ mT, where we assume an uncertainty of 1 μm for the width of the stripe. The increased contribution from interface B_{FL} in thick YIG films may be attributed to the slight increase in interface quality (Section S4). The small negative B_{FL} inferred from our measurements with Pt as overlayer is consistent with harmonic torque measurements reported in Ref.[17] for thick YIG films.

S6. Analysis of $R^{2\omega}_{xy,\mathrm{SSE}}$

Current and thickness dependence

The spin Seebeck effect (SSE) appears in the $\cos\phi$ term in angle-dependent second harmonic Hall measurements $R^{2\omega}_{xy}(\phi)$ [Eq. (3) of the main text]. Our measurements show that $R^{2\omega}_{xy,\mathrm{SSE}}$ scales linearly with the applied current (Fig. S5b), consistent with an increase in the temperature gradient due to Joule heating. Furthermore, we have estimated the SSE for all investigated thicknesses and observed that the effect diminishes with decreasing thickness (Fig. S5a), as expected[14].

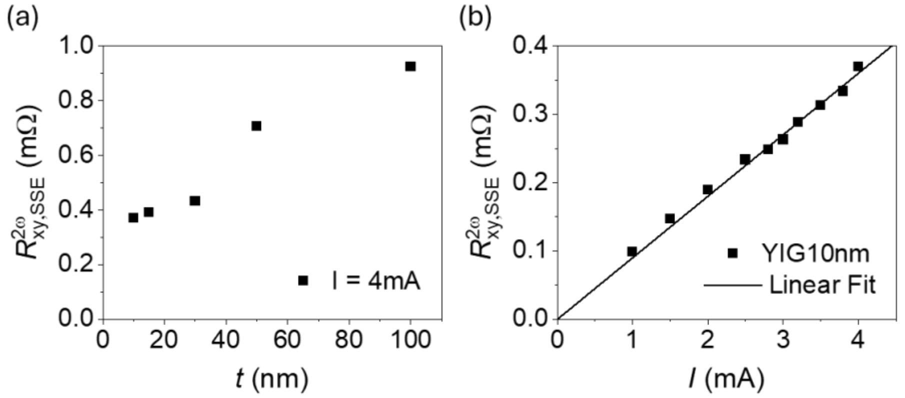

Figure S5. Variation of $R^{2\omega}_{xy,\mathrm{SSE}}(B = 1\ \mathrm{T})$ as a function of (a) thickness and (b) current. $R^{2\omega}_{xy,\mathrm{SSE}}$ is estimated from the $R^{2\omega}_{xy}(\phi)$ as explained in Section S5. The solid line represents the linear fit to the data. Error bars in (a) and (b), which are smaller than the size of the dots, were estimated from the uncertainty of the data fits to $R^{1\omega}_{xy}(\phi)$ and $R^{2\omega}_{xy}(\phi)$ and error propagation.

Field dependence

The analysis of $R^{2\omega}_{xy}(\phi)$ reveals a field dependent spin Seebeck effect (SSE) contribution (Fig. 2a of the main text). We found that this field dependence is consistent with $R^{1\omega}_{xy,\mathrm{SMR}} \propto M_{\parallel}^2(B)$ (Fig. S6a), where $M_{\parallel}$ denotes the magnetic component collinear with the magnetic field [see Section S9 for more details regarding $M_{\parallel}(B)$]. The field dependence of the SSE and its direct correlation with $R^{1\omega}_{xy,\mathrm{SMR}}$ (Fig. S6a) is

explained as follows. The SSE amplitude is known to correlate with g_r[18,19]. Considering $M_\parallel \sim M_s$, g_r relates to $M_\parallel$ as $g_r \propto \langle M_\parallel^2(B)\rangle$[9,20]. Therefore $R^{2\omega}_{xy,\mathrm{SSE}} \propto M_\parallel^2(B) \propto R^{1\omega}_{xy,\mathrm{SMR}}$ as demonstrated in Fig. S6a. Our analysis shows that the field dependence of $R^{2\omega}_{xy,\mathrm{SSE}}$ in thin films is dominated by the gradual evolution of $M(B)$ towards M_s (Fig. S6a). Similar behaviour was found across all samples explored in this study. For thicker YIG layers, however, one also needs to take into account that magnons relax across the thickness, which results in an additional field dependence to $R^{2\omega}_{xy,\mathrm{SSE}}$[13].

Additionally, we performed field-dependent SSE measurements by externally inducing a vertical thermal gradient across the YIG/Pt interface. As example, here we present data taken in 30 nm YIG where the SSE signal is sufficiently large to be explored in non-local experiments by using devices with injector-detector distances much larger than the magnon diffusion length (in 30nm-thick YIG we found $\lambda_m \sim 0.5$ µm[21]). In such scenario, thermally-driven magnon signals induced by ac currents applied to the injector become negligible, and only the thermoelectric signals arising from the thermal gradients at the detector are captured in second harmonic transport measurements[22]. Figure S6b shows the SSE signal detected in a non-local device structure with $d = 8.7$ µm and the magnetic field applied collinear with the spin accumulation at the injector (y direction, see sketch). Hence, $M(B) \parallel y$ and the SSE signal $R^{2\omega}_{\mathrm{NL}-\nabla\mathbf{T}_z}(B)$ is proportional to the out-of-plane thermal gradient $\nabla\mathbf{T}_z$. Note that this configuration is equivalent to the one used to extract $R^{2\omega}_{\mathrm{xy,SSE}}(B)$ in harmonic analysis. Within the error associated in the measurement, which is in the limit of the technique, we find a reasonable agreement between $R^{2\omega}_{\mathrm{NL}-\nabla\mathbf{T}_z}(B)$ (black dots) and $R^{2\omega}_{\mathrm{xy,SSE}}$ (red dots) (Fig. S6b).

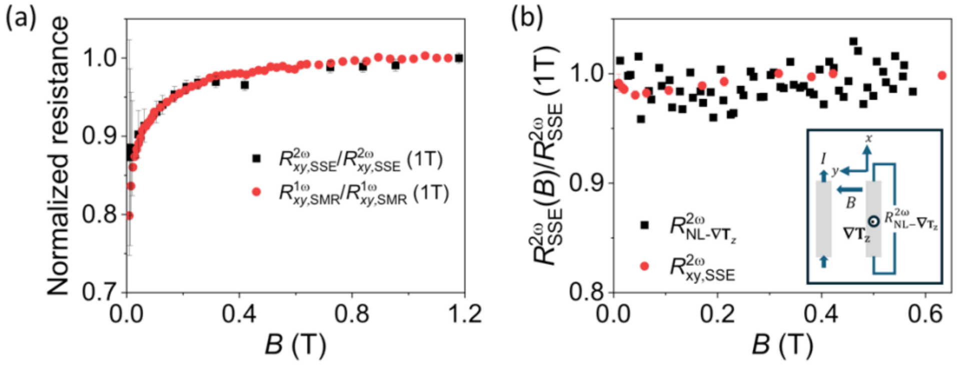

Figure S6. (a) Comparison of $R^{2\omega}_{xy,\mathrm{SSE}}(B)$ (black dots) and $R^{1\omega}_{xy,\mathrm{SMR}}(B)$ (red dots) for 10 nm YIG at 4 mA. The data is normalized by the values at $B = 1$ T. The error bars are estimated from the uncertainty of the angular-dependent fits and error propagation. For $R^{1\omega}_{xy,\mathrm{SMR}}$, the errors are smaller than the dots size. (b) SSE measurements performed in YIG(30nm)/Pt (black dots) by employing a non-local device structure as the one shown in the sketch. The width of the stripes is 500 nm, and the center-to-center distance between injector and detector $d = 8.7$ µm. The red points present the $R^{2\omega}_{xy,\mathrm{SSE}}$ data obtained from harmonic Hall measurements in YIG(30nm)/Pt using Eq. (3). Data normalized at 1 T values for comparison.

S7. Non-linear SMR from longitudinal $R^{2\omega}_{xx}$ measurements

In the main text, we focused on harmonic Hall measurements to evaluate $\Delta M/M_s$. These measurements are more convenient due to the higher signal-to-noise ratio. Nevertheless, magnon creation/annihilation processes also result in a non-linear magnetoresistance response in the longitudinal configuration. In YIG/Pt, all relevant contributions to $R^{2\omega}_{xx}(\phi)$ read as[11]

$$R_{xx}^{2\omega}(\phi) = R_{\sin}^{2\omega}\sin\phi + R_{\sin^3}^{2\omega}\sin^3\phi$$

$$= (2R_{xx,\mathrm{FL}}^{2\omega} + R_{xx,\mathrm{SSE}}^{2\omega})\sin\phi + (R_{xx,\mathrm{SMR}}^{2\omega} - 2R_{xx,\mathrm{FL}}^{2\omega})\sin^3\phi, \quad \text{(S2)}$$

where $R_{xx,\mathrm{FL}}^{2\omega}$, $R_{xx,\mathrm{SSE}}^{2\omega}$, and $R_{xx,\mathrm{SMR}}^{2\omega}$ refer to the longitudinal counterpart of the FL torque, SSE, and non-linear SMR contributions, respectively. They relate to the transverse components by the geometrical factor $g = L/w \approx 10$ as $R_{xx,\mathrm{i}}^{2\omega} \approx -gR_{xy,\mathrm{i}}^{2\omega}$ ($i = \mathrm{FL}, \mathrm{SSE}, \text{or SMR}$)[11,16]. Note that the weights of the $R_{xx,\mathrm{i}}^{2\omega}$ components to the $\sin\phi$ and $\sin^3\phi$ terms [Eq. (S2)] are different than the weights of the $R_{xy,\mathrm{i}}^{2\omega}$ components to the terms in Eq. (3). This makes possible to independently evaluate ΔM from longitudinal measurements using $\Delta M = R_{xx,\mathrm{SMR}}^{2\omega}/2R_{xx,\mathrm{SMR}}^{1\omega}$. Here $R_{xx,\mathrm{SMR}}^{1\omega}$ stands for the SMR amplitude measured in the first harmonic longitudinal resistance as $R_{xx}^{1\omega}(\phi) = R_0 - R_{xx,\mathrm{SMR}}^{1\omega}\sin^2\phi$, with R_0 a base resistance, and relates to the transverse SMR as $R_{xx,\mathrm{SMR}}^{1\omega} = gR_{xy,\mathrm{SMR}}^{1\omega}$.

Figure S7a presents the field dependence of $R_{\sin}^{2\omega}$ and $R_{\sin^3}^{2\omega}$ in YIG(10nm)/Pt extracted from $R_{xx}^{2\omega}(\phi)$ data [Eq. (S2)]. To accurately calculate $R_{xx,\mathrm{SMR}}^{2\omega}$ we use the $R_{\mathrm{xy}}^{2\omega}$ data (Fig. 2a of the main text) to estimate the SSE and FL contributions. The comparison of the non-linear SMR amplitudes is presented in Figure S7b showing a good agreement with each other, confirming that $\Delta M/M_{\mathrm{s}}$ arising from magnon creation/annihilation processes can be extracted from longitudinal measurements as well.

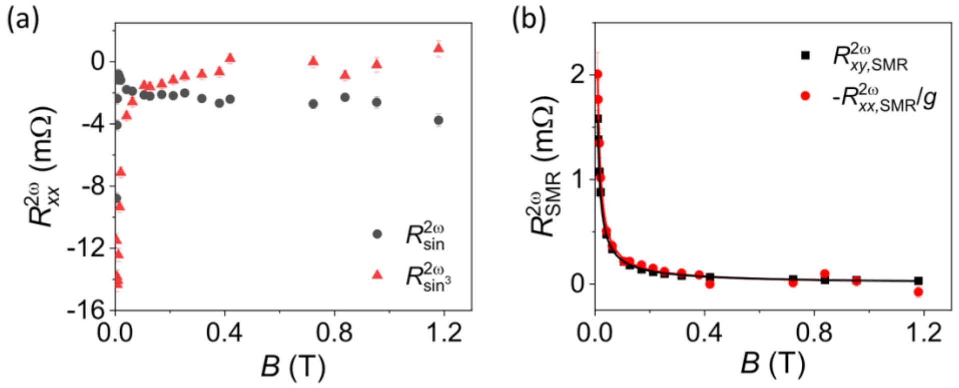

Figure S7. (a) Field dependence of the $R_{\sin}^{2\omega}$ (black dots) and $R_{\sin^3}^{2\omega}$ (red triangles) contributions to $R_{xx}^{2\omega}(\phi)$ [Eq. (S2)] in YIG(10nm)/Pt measured at $I = 4$ mA. Error bars represent the uncertainty associated to the fit of the angular scan data to Eq. (S2). (b) Comparison of the field dependence of the longitudinal and transverse non-linear SMR. $R_{xx,\mathrm{SMR}}^{2\omega}(B)$ is calculated from panel (a) using Eq. (S2). $R_{xy,\mathrm{SMR}}^{2\omega}$ is extracted from Fig. 2a of the main text. The solid line is the fit of the data to $B^{-\gamma}$. Error bars are calculated from error propagation.

S8. The role of stiffness in the field dependence of ΔM

The magnon stiffness D can affect ΔM via the relaxation time τ ($\Delta M \propto \tau$; see derivation of Eq. (4) in the main text, where $I_{\mathrm{c}} = 1/\varepsilon\beta\tau$). For high frequency exchange magnons, τ is well described by[23] $\tau(\omega) = 1/2\alpha\omega$ with $\omega = Dk^2$. For low frequencies, which is the dominant regime in our experiments, the dispersion relation needs to be modified to account for the magnetostatic energy, which is described by[24] $\omega(k) = \sqrt{(\omega_0 + Dk^2)(\omega_0 + Dk^2 + F\omega_{\mathrm{M}})}$, where $\omega_0 = \gamma_{\mathrm{m}}\mu_0 H$, $\omega_{\mathrm{M}} = \gamma_{\mathrm{m}}\mu_0 M_{\mathrm{s}}$, and F is a factor that depends on the relative orientation between $\mathbf{k}$ and $\mathbf{M}$. Considering a randomly distributed $\mathbf{k}$, the magnon dispersion can be approximated by

$$\omega(k) \approx \sqrt{(\omega_0 + Dk^2)(\omega_0 + Dk^2 + \omega_{\mathrm{M}}/2)}\,. \quad \text{(S3)}$$

We can see that for large k values the dispersion recovers the expected $\omega(k) \approx Dk^2$ dependence with no influence by magnetic field. For $k \to 0$, which is the relevant case in our experiments, and using Eq. (S3) we find that τ follows a dependence of the form $\tau_0/(1+\xi B)$, which is in agreement with experiments[22,25]. Here ξ is a constant that depends on D, γ_{m}, and M_{s}, and τ_0 is the relaxation time at $B=0$. The reduction of τ with field mimics the increase of the effective Gilbert damping with the magnetic field via ξ.

In the linear regime for I, and using Eq. (4) of the main text, the expected field dependence of ΔM due to the modulation of the low frequency magnons via $\tau(B)$ is given by $\Delta M \propto 1/(1+\xi B)$, which is not consistent with the $B^{-\gamma}$ dependence inferred from experiments (Figs. 4a,b of the main text). Fit of the experimental data to a $1/(1+\xi B)$ dependence in the full field range up to $B{\sim}1$ T results in poor regression fits, indicating that the dominant mechanism for the suppression of ΔM with field is not $\tau(B)$.

S9. Field dependence of $\Delta R^{1\omega}_{xy,\mathrm{SMR}}$

In the main text, we show that the relative change in the magnetization of the YIG layer follows a power law with the magnetic field (Fig. 4). As discussed in the main text and Section S8, we cannot attribute the observations to the effect of D on $\Delta M(B)$. Alternatively, we can qualitatively explain our results by considering that the spin conductance g_{s} is the dominant parameter controlling $\Delta M(B)$. This argument is based on the field dependence of the linear magnetoresistance $R^{1\omega}_{xy,\mathrm{SMR}}$ as follows.

The microscopic theory describing the spin transmission across heavy metal/magnetic insulator interfaces predicts that g_{s} is $\propto \langle M_\perp^2\rangle$[9,20]. This relation is valid in the limit $k \to 0$, which is the case relevant in our experiments, and has been experimentally verified in paramagnetic insulators[26]. On the other hand, $R^{1\omega}_{xy,\mathrm{SMR}}$ describes the amplitude of the magnetization that is collinear to the external magnetic field, $M_\parallel$, following $R^{1\omega}_{xy,\mathrm{SMR}} \propto M_\parallel^2$. Note that in YIG, the in-plane crystal anisotropy is in the μT range[1], making the in-plane Hall resistance to follow a $R^{1\omega}_{xy} \approx R^{1\omega}_{xy,\mathrm{SMR}} \cos\phi \sin\phi$ angle dependence for B as small as few mT. Considering M_{s} the saturation magnetization, i.e., M in the limit $B \to \infty$, we can define $M_{\mathrm{s}}^2 = M_\parallel^2 + M_\perp^2$, with $M_\perp$ describing the transverse component of the magnetization. In this regard, we can define the field dependence of the magnetoresistance as

$$R^{1\omega}_{xy,\mathrm{SMR}}(B) = R^{1\omega,\mathrm{sat}}_{xy,\mathrm{SMR}} - \Delta R^{1\omega}_{xy,\mathrm{SMR}}(B), \qquad \text{(S4)}$$

where $R^{1\omega,\mathrm{sat}}_{xy,\mathrm{SMR}} \propto M_{\mathrm{s}}^2$ describes the maxima of the magnetoresistance amplitude when the magnetization is fully saturated along B. Accordingly, $\Delta R^{1\omega}_{xy,\mathrm{SMR}}(B)$ captures the modulation of the magnetoresistance associated to the suppression of magnetic fluctuations with magnetic field. Therefore, in a first approximation, $\Delta R^{1\omega}_{xy,\mathrm{SMR}}(B)$ is expected to be related to $\langle M_\perp^2\rangle(B)$ in analogy to the quadratic dependence of the magnetization of the other two terms in Eq. (S4). As a result, $\Delta R^{1\omega}_{xy,\mathrm{SMR}}(B)$, which can be experimentally evaluated by performing field dependent $R^{1\omega}_{xy,\mathrm{SMR}}$ measurements, can be used to estimate $g_{\mathrm{s}}(B)$ due to their mutual dependence on $\langle M_\perp^2\rangle$. The analysis of $R^{1\omega}_{xy,\mathrm{SMR}}(B)$ reveals that $\Delta R^{1\omega}_{xy,\mathrm{SMR}}(B)$ follows a power law with the magnetic field $B^{-\eta}$ (Fig. S8) as empirically determined for $\Delta M(B)$ (Fig. 4). Experiments performed for different YIG thickness (Fig. S8a) and currents (Fig. S8b) show that η evolves with t and I in the same fashion as γ does (Tables S4,S5 and Fig. 4c), strongly indicating that $\Delta M(B)$ is described by the suppression of g_{s} with field. Remarkably, the η values determined from $\Delta R^{1\omega}_{xy,\mathrm{SMR}}(B)$ are quantitatively closely related to the γ values found for $\Delta M(B)$ in thick films, whereas for thin films the

values deviate. Nevertheless, $\Delta M(B)$ can also be influenced by other terms such as $\tau(B)$ and $\mu_{\rm m}(B)$, which could explain the quantitative discrepancy between η and γ. A more detailed theoretical analysis, which is beyond the scope of this work, is required to quantitatively account for the differences in η and γ values and identify whether other parameters beyond $g_{\rm s}(B)$ might also significantly contribute to $\Delta M(B)$.

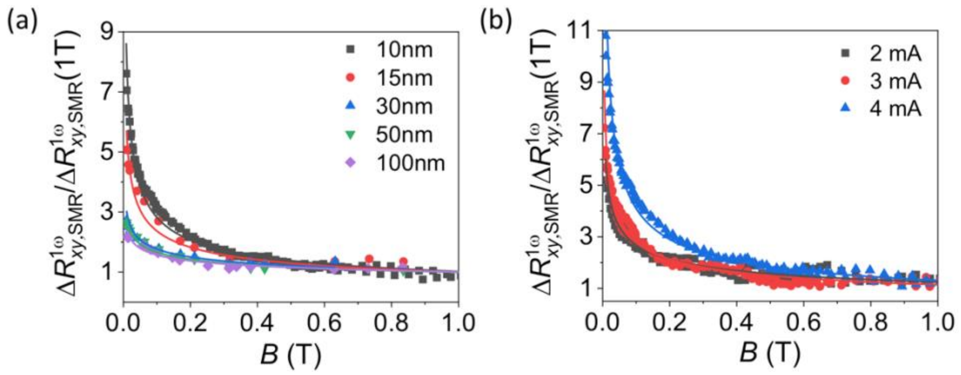

Figure S8. (a) and (b), Magnetic field dependence of $\Delta R^{1\omega}_{xy,\rm SMR}$ evaluated from $R^{1\omega}_{xy}(B)$ measurements (data points) taken for different YIG thicknesses ($I = 4$mA) and various currents ($t = 10$nm), respectively. The data is normalized by the $\Delta R^{1\omega}_{xy,\rm SMR}$ value at 1 T for comparison. The solid lines are data fits to $B^{-\eta}$, revealing that η decreases with the thickness and grows with the current (Tables S4 and S5).

YIG thickness (nm)	η	γ
10	0.47±0.01	0.83±0.01
15	0.37±0.03	0.52±0.02
30	0.23±0.02	0.34±0.01
50	0.22±0.01	0.26±0.01
100	0.19±0.02	0.25±0.03

Table S4. η and γ values determined from $\Delta R^{1\omega}_{xy,\rm SMR}(B)$ and $R^{2\omega}_{xy,\rm SMR}(B)$ measurements, respectively, for different YIG thicknesses at $I =$ 4 mA. The error bars represent the variance of the experimental data points to the fit functions.

I (mA)	η
2	0.33±0.01
3	0.39±0.01
4	0.47±0.01

Table S5. η values determined from $\Delta R^{1\omega}_{xy,\rm SMR}$ measurements at different currents in 10 nm YIG. The error bars represent the variance of the experimental data points to the fit functions.

S10. Estimation of damping values of our YIG films

The Gilbert damping was evaluated in YIG(15nm)/Pt(4nm) and YIG(50nm)/Pt(4nm) via ferromagnetic resonance (FMR) measurements in sister samples. The values for the other stacks were estimated by considering following scaling for the damping[27]

$$\alpha = \alpha_0 + \frac{\alpha'}{t} + \frac{\alpha''}{t^2} \approx \frac{\alpha'}{t}, \qquad \text{(S5)}$$

where α_0 is the bulk value, which is in the range $10^{-5} - 10^{-4}$, and α' and α'' are two empirical constants. The $1/t^2$ term comes from the vertical confinement of the YIG layer, whereas the $1/t$ accounts for the interface-induced increase of the damping due to the spin-orbit coupling of Pt. To leading order, the damping of our YIG films can be well approximated by $\alpha \approx \frac{\alpha'}{t}$. Figure S9 shows the measured α in YIG(15nm)/Pt(4nm) and YIG(50nm)/Pt(4nm) (black triangles), reference literature values obtained from FMR measurements in similar YIG/Pt stacks[27,28], and the fit of all data points to Eq. (S5) with $\alpha' = (119 \pm 3) \cdot 10^{-3}$ nm. The α values for our YIG(t)/Pt samples with thickness $t = 10, 30,$ and 100 nm are

estimated from the fit (green stars in Fig. S9). Table S6 summarizes the α values used in the analysis of Fig. 5b of the manuscript.

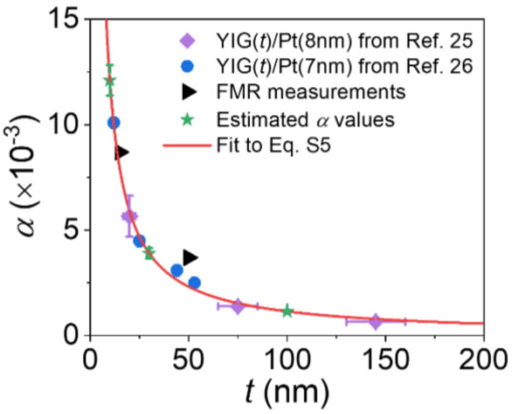

Figure S9. Gilbert damping values for YIG/Pt. Black triangles are the values measured in YIG(15nm)/Pt(4nm) and YIG(50nm)/ Pt(4nm) via FMR. Purple and blue dots are reference literature values extracted from Refs.[27] and[28]. Green stars are estimated α values for YIG(t)/Pt of thicknesses 10, 30 and 100 nm extracted from fitting the data points to Eq. (S5) (red line). The nominal YIG thickness values are the ones considered following literature convention.

Thickness (nm)	$\boldsymbol{\alpha}$
10	$12.1 \pm 1.3 \times 10^{-3}$
**15**	$\mathbf{8.7 \pm 0.2 \times 10^{-3}}$
30	$3.9 \pm 0.4 \times 10^{-3}$
**50**	$\mathbf{3.7 \pm 0.1 \times 10^{-3}}$
100	$1.1 \pm 0.1 \times 10^{-3}$

Table S6. Gilbert damping values for YIG(15nm)/Pt(4nm) and YIG(50nm)/Pt(4nm) evaluated via FMR measurements (bolted values) and estimates for the other YIG(t)/Pt samples according to Eq. (S5) (Fig. S9).

S11. Joule heating and its effect on $\Delta M/M_s$ and $j_{s1}^{\text{int}}/j_{s2}^{\text{int}}$

The application of current through the Pt layer induces Joule heating, leading to a reduction in M_s. As seen In Eq. (5), the only factor that may affect the calculation of $j_{s1}^{\text{int}}/j_{s2}^{\text{int}}$ is the ratio M_{s2}/M_{s1}, which can be estimated according to the data presented in Section S1.

To quantify the increase of temperature due to Joule heating, we performed temperature-dependent resistance measurements $R(T)$ on a Pt device in our YIG films (by using a Peltier stage and applying small current to the device to avoid Joule heating), and compare it with the resistance measured when applying large currents to the same device. The calibrated $R(T)$ is presented in Fig. S10. From transport measurements in our devices we found that a heating of ~9 K is obtained for an ac current $I = 4$ mA, which is in the range of the largest currents employed in our experiments. This increase in temperature results in a reduction of M_s. Accordingly, the ratio $\left(\frac{M_{s2}}{M_{s1}}\right)_{302\text{K}}$ increases by a maximum of 7% for 10nm YIG compared to 100nm at room temperature, i.e., 293 K (see Table S7). Note that the upturn of $\Delta M/M_s$ observed in Fig. 5a at 4mA corresponds to an increase of $> 400\%$ compared to the value expected from extrapolating the linear regime of $\Delta M(I)/M_s$. This shows that heating cannot explain the large enhancement of $\Delta M/M_s$ with current for 10 nm YIG (Figs. 3a and 5a), further highlighting the key role of current-induced damping compensation by spin-flip scattering processes in our experiments. Besides, $j_{s1}^{\text{int}}/j_{s2}^{\text{int}}$ was evaluated from the asymptotic trend of $\Delta M/M_s$ in the small current regime ($I < 2$ mA), where temperature changes by Joule heating are < 2 K and thus comparable to temperature variations in the laboratory. Therefore, Joule heating was neglected, and the $\left(\frac{M_{s2}}{M_{s1}}\right)_{293K}$ values were the ones used to estimate $j_{s1}^{\text{int}}/j_{s2}^{\text{int}}$ in Fig. 5b.

FIG S10. Pt resistance measured as a function of temperature. The solid line is a linear fit to the data.

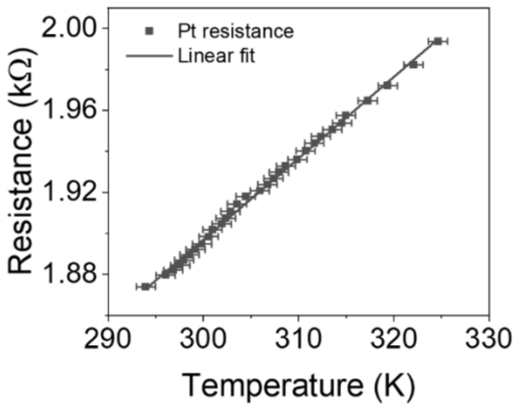

t (nm)	$\frac{M_{s,100}}{M_{s,t}}$ at 302K	$\frac{M_{s,100}}{M_{s,t}}$ at 293K
10	1.66 ± 0.33	1.55 ± 0.31
15	1.43 ± 0.14	1.35 ± 0.13
30	1.18 ± 0.05	1.12 ± 0.05
50	1.11 ± 0.03	1.10 ± 0.02
100	1	1

Table S7. M_{s2}/M_{s1} factors calculated for different YIG thicknesses and temperatures taking the value for 100 nm as reference 2.

S12. The highest occupied magnon band for different t and T^{eff}

The highest occupied magnon band N is given by[29]

$$N = \mathrm{int}\left(\frac{t_{\mathrm{YIG}}}{\pi}\sqrt{\frac{k_B T^{\mathrm{eff}}}{\hbar\gamma_{\mathrm{m}} D}}\right). \qquad \text{(S6)}$$

Consequently, the number of vertical k_z modes for a given magnon with energy $\varepsilon_k = D\hbar\gamma_{\mathrm{m}}k^2 = k_{\mathrm{B}}T^{\mathrm{eff}}$ is $N+1$, where $k^2 = k_z^2 + k_\parallel^2$ is the wavevector of the magnon with $\mathbf{k}_\parallel = (k_x, k_y)$ the in plane component. As can be seen in Figure S11, the number of occupied k_z magnon bands reduces to just a few when T^{eff} reduces to the Kelvin regime. Concretely, at $T^{\mathrm{eff}} \sim 10K$, $N+1 = 13$ for 100nm YIG whereas for 10 nm YIG it reduces to 2. At $T^{\mathrm{eff}} < 6.6$ K, only the fundamental $k_z = 0$ magnon band is occupied for 10 nm YIG.

Note that the mean field theory predicts that the spin wave stiffness D scales linearly with the Curie temperature T_{c}. In our 10 nm film we found that T_{c} decreases to $\sim$354 K (Section S1), which is about 1.6 times smaller than the bulk value. Taking into account the expected reduction compared to the bulk value, the number of occupied k_z modes at $T^{\mathrm{eff}} = 10$ K for a 10 nm film remains the same with $N+1 = 2$ due to its $D^{-1/2}$ dependence. This shows that the qualitative picture presented here for magnon band occupation is robust to small gradual variations in D across the samples.

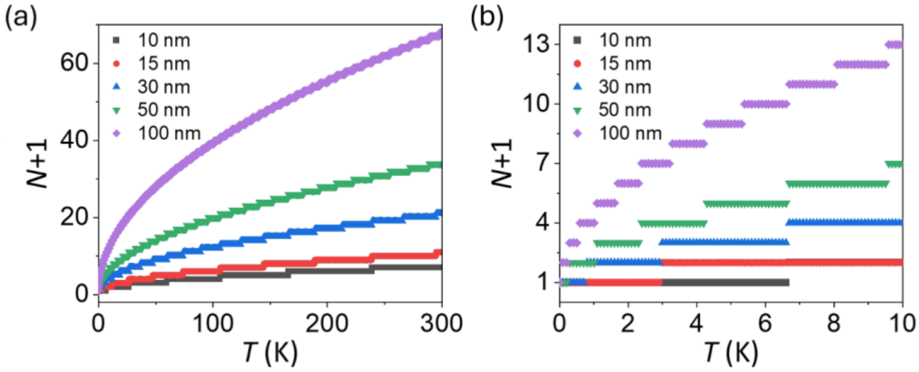

Figure S11. (a) The number of occupied perpendicular standing spin wave k_z bands at different temperatures and for various YIG thicknesses calculated from Eq. (S6) with $D = 5 \times 10^{-17}$ Tm^2[30]. (b) Same as panel (a) in the subthermal regime <10 K.

References

(1) Mendil, J.; Trassin, M.; Bu, Q.; Schaab, J.; Baumgartner, M.; Murer, C.; Dao, P. T.; Vijayakumar, J.; Bracher, D.; Bouillet, C.; Vaz, C. A. F.; Fiebig, M.; Gambardella, P. Magnetic Properties and Domain Structure of Ultrathin Yttrium Iron Garnet/Pt Bilayers. *Phys. Rev. Mater.* **2019**, *3* (3), 034403. https://doi.org/10.1103/PhysRevMaterials.3.034403.

(2) Mitra, A.; Cespedes, O.; Ramasse, Q.; Ali, M.; Marmion, S.; Ward, M.; Brydson, R. M. D.; Kinane, C. J.; Cooper, J. F. K.; Langridge, S.; Hickey, B. J. Interfacial Origin of the Magnetisation Suppression of Thin Film Yttrium Iron Garnet. *Sci. Rep.* **2017**, *7* (1), 11774. https://doi.org/10.1038/s41598-017-10281-6.

(3) Shao, Q.; Tang, C.; Yu, G.; Navabi, A.; Wu, H.; He, C.; Li, J.; Upadhyaya, P.; Zhang, P.; Razavi, S. A.; He, Q. L.; Liu, Y.; Yang, P.; Kim, S. K.; Zheng, C.; Liu, Y.; Pan, L.; Lake, R. K.; Han, X.; Tserkovnyak, Y.; Shi, J.; Wang, K. L. Role of Dimensional Crossover on Spin-Orbit Torque Efficiency in Magnetic Insulator Thin Films. *Nat. Commun.* **2018**, *9*, 3612. https://doi.org/10.1038/s41467-018-06059-7.

(4) Cooper, J. F. K.; Kinane, C. J.; Langridge, S.; Ali, M.; Hickey, B. J.; Niizeki, T.; Uchida, K.; Saitoh, E.; Ambaye, H.; Glavic, A. Unexpected Structural and Magnetic Depth Dependence of YIG Thin Films. *Phys. Rev. B* **2017**, *96* (10), 104404. https://doi.org/10.1103/PhysRevB.96.104404.

(5) Vélez, S.; Schaab, J.; Wörnle, M. S. S.; Müller, M.; Gradauskaite, E.; Welter, P.; Gutgsell, C.; Nistor, C.; Degen, C. L. L.; Trassin, M.; Fiebig, M.; Gambardella, P. High-Speed Domain Wall Racetracks in a Magnetic Insulator. *Nat. Commun.* **2019**, *10* (1), 4750. https://doi.org/10.1038/s41467-019-12676-7.

(6) Uchida, K.; Kikkawa, T.; Miura, A.; Shiomi, J.; Saitoh, E. Quantitative Temperature Dependence of Longitudinal Spin Seebeck Effect at High Temperatures. *Phys. Rev. X* **2014**, *4* (4), 041023. https://doi.org/10.1103/PhysRevX.4.041023.

(7) Vaz, C. A. F.; Bland, J. A. C.; Lauhoff, G. Magnetism in Ultrathin Film Structures. *Reports Prog. Phys.* **2008**, *71*, 056501.

(8) Althammer, M.; Meyer, S.; Nakayama, H.; Schreier, M.; Altmannshofer, S.; Weiler, M.; Huebl, H.; Geprägs, S.; Opel, M.; Gross, R.; Meier, D.; Klewe, C.; Kuschel, T.; Schmalhorst, J.-M.; Reiss, G.; Shen, L.; Gupta, A.; Chen, Y.-T.; Bauer, G. E. W.; Saitoh, E.; Goennenwein, S. T. B. Quantitative Study of the Spin Hall Magnetoresistance in Ferromagnetic Insulator/Normal Metal Hybrids. *Phys. Rev. B* **2013**, *87* (22), 224401. https://doi.org/10.1103/PhysRevB.87.224401.

(9) Vélez, S.; Golovach, V. N.; Gomez-Perez, J. M.; Chuvilin, A.; Bui, C. T.; Rivadulla, F.; Hueso, L. E.; Bergeret, F. S.; Casanova, F. Spin Hall Magnetoresistance in a Low-Dimensional Heisenberg Ferromagnet. *Phys. Rev. B* **2019**, *100* (18), 180401. https://doi.org/10.1103/PhysRevB.100.180401.

(10) Cornelissen, L. J.; Peters, K. J. H.; Bauer, G. E. W.; Duine, R. A.; Van Wees, B. J. Magnon Spin Transport Driven by the Magnon Chemical Potential in a Magnetic Insulator. *Phys. Rev. B* **2016**, *94* (1), 014412. https://doi.org/10.1103/PhysRevB.94.014412.

(11) Noel, P.; Schlitz, R.; Karadza, E.; Lambert, C.-H.; Nessi, L.; Binda, F.; Gambardella, P. Nonlinear Longitudinal and Transverse Magnetoresistances Due to Magnon Creation-Annihilation Processes. *Phys. Rev. Lett.* **2025**, *134*, 146701.

(12) Noël, P.; Karadža, E.; Schlitz, R.; Welter, P.; Lambert, C.-H.; Nessi, L.; Binda, F.; Degen, C. L.; Gambardella, P. Estimation of Spin-Orbit Torques in the Presence of Current-Induced Magnon Creation and Annihilation. *Phys. Rev. B* **2024**, *111* (14), 144409. https://doi.org/10.1103/PhysRevB.111.144409.

(13) Ritzmann, U.; Hinzke, D.; Kehlberger, A.; Guo, E. J.; Kläui, M.; Nowak, U. Magnetic Field Control of the Spin Seebeck Effect. *Phys. Rev. B* **2015**, *92* (17), 174411. https://doi.org/10.1103/PhysRevB.92.174411.

(14) Kehlberger, A.; Ritzmann, U.; Hinzke, D.; Guo, E. J.; Cramer, J.; Jakob, G.; Onbasli, M. C.; Kim, D. H.; Ross, C. A.; Jungfleisch, M. B.; Hillebrands, B.; Nowak, U.; Kläui, M. Length Scale of the Spin Seebeck Effect. *Phys. Rev. Lett.* **2015**, *115* (9), 096602. https://doi.org/10.1103/PhysRevLett.115.096602.

(15) Kikkawa, T.; Uchida, K.-I.; Daimon, S.; Qiu, Z.; Shiomi, Y.; Saitoh, E. Critical Suppression of Spin Seebeck Effect by Magnetic Fields. *Phys. Rev. B* **2015**, *92* (6), 064413. https://doi.org/10.1103/PhysRevB.92.064413.

(16) Manchon, A.; Miron, I. M.; Jungwirth, T.; Sinova, J.; Zelezny, J.; Thiaville, A.; Garello, K.; Gambardella, P. Current-Induced Spin-Orbit Torques in Ferromagnetic and Antiferromagnetic Systems. *Rev. Mod. Phys.* **2019**, *91*, 035004.

(17) Mendil, J.; Trassin, M.; Bu, Q.; Fiebig, M.; Gambardella, P. Current-Induced Switching of YIG/Pt Bilayers with in-Plane Magnetization Due to Oersted Fields. *Appl. Phys. Lett.* **2019**, *114* (17), 172404. https://doi.org/10.1063/1.5090205.

(18) Xiao, J.; Bauer, G. E. W.; Uchida, K.-I.; Saitoh, E.; Maekawa, S. Theory of Magnon-Driven Spin Seebeck Effect. *Phys. Rev. B* **2010**, *81*, 214418.

(19) Weiler, M.; Althammer, M.; Schreier, M.; Lotze, J.; Meyer, S.; Huebl, H.; Gross, R.; Kamra, A.; Xiao, J.; Chen, Y.; Jiao, H.; Bauer, G. E. W.; Goennenwein, S. T. B. Experimental Test of the Spin Mixing Interface Conductivity Concept. *Phys. Rev. Lett.* **2013**, *111* (1), 176601.

(20) Zhang, X.-P.; Bergeret, F. S.; Golovach, V. N. Theory of Spin Hall Magnetoresistance from a Microscopic Perspective. *Nano Lett.* **2019**, *19* (9), 6330–6337. https://doi.org/10.1021/acs.nanolett.9b02459.

(21) Schlitz, R.; Vélez, S.; Kamra, A.; Lambert, C.-H.; Lammel, M.; Goennenwein, S. T. B.; Gambardella, P. Control of Nonlocal Magnon Spin Transport via Magnon Drift Currents. *Phys. Rev. Lett.* **2021**, *126* (25), 257201. https://doi.org/10.1103/physrevlett.126.257201.

(22) Gao, J.; Lambert, C. H.; Schlitz, R.; Fiebig, M.; Gambardella, P.; Vélez, S. Magnon Transport and Thermoelectric Effects in Ultrathin Tm3Fe5O12/Pt Nonlocal Devices. *Phys. Rev. Res.* **2022**, *4* (4), 043214. https://doi.org/10.1103/PhysRevResearch.4.043214.

(23) Borisenko, I. V.; Demidov, V. E.; Urazhdin, S.; Rinkevich, A. B.; Demokritov, S. O. Relation between Unidirectional Spin Hall Magnetoresistance and Spin Current-Driven Magnon Generation. *Appl. Phys. Lett.* **2018**, *113* (6), 062403. https://doi.org/10.1063/1.5044737.

(24) Mahmoud, A.; Ciubotaru, F.; Vanderveken, F.; Chumak, A. V.; Hamdioui, S.; Adelmann, C.; Cotofana, S. Introduction to Spin Wave Computing. *J. Appl. Phys.* **2020**, *128* (16), 161101. https://doi.org/10.1063/5.0019328.

(25) Gückelhorn, J.; Wimmer, T.; Müller, M.; Geprägs, S.; Hübl, H.; Gross, R.; Althammer, M. Magnon Transport in Y3Fe5O12/Pt Nanostructures with Reduced Effective Magnetization. *Phys. Rev. B* **2021**, *104*, L180410.

(26) Oyanagi, K.; Gomez-Perez, J. M.; Zhang, X.-P.; Kikkawa, T.; Chen, Y.; Sagasta, E.; Chuvilin, A.; Hueso, L. E.; Golovach, V. N.; Bergeret, F. S.; Casanova, F.; Saitoh, E. Paramagnetic Spin Hall Magnetoresistance. *Phys. Rev. B* **2021**, *104* (13), 134428. https://doi.org/10.1103/PhysRevB.104.134428.

(27) Jungfleisch, M. B.; Chumak, A. V.; Kehlberger, A.; Lauer, V.; Kim, D. H.; Onbasli, M. C.; Ross, C. A.; Kläui, M.; Hillebrands, B. Thickness and Power Dependence of the Spin-Pumping Effect in Y3Fe5 O12 /Pt Heterostructures Measured by the Inverse Spin Hall Effect. *Phys. Rev. B* **2015**, *91* (13), 134407. https://doi.org/10.1103/PhysRevB.91.134407.

(28) Haertinger, M.; Back, C. H.; Lotze, J.; Weiler, M.; Geprägs, S.; Huebl, H.; Goennenwein, S. T. B.; Woltersdorf, G. Spin Pumping in YIG/Pt Bilayers as a Function of Layer Thickness. *Phys. Rev. B* **2015**, *92* (5), 054437. https://doi.org/10.1103/PhysRevB.92.054437.

(29) Wei, X. Y.; Santos, O. A.; Lusero, C. H. S.; Bauer, G. E. W.; Ben Youssef, J.; van Wees, B. J. Giant Magnon Spin Conductivity in Ultrathin Yttrium Iron Garnet Films. *Nat. Mater.* **2022**, *21* (12), 1352–1356. https://doi.org/10.1038/s41563-022-01369-0.

(30) Klingler, S.; Chumak, A.; Mewes, T.; Khodadadi, B.; Mewes, C.; Dubs, C.; Surzhenko, O.; Hillebrands, B.; Conca, A. Measurements of the Exchange Stiffness of YIG Films Using Broadband Ferromagnetic Resonance Techniques. *J. Phys. D. Appl. Phys.* **2015**, *48* (1), 015001. https://doi.org/10.1088/0022-3727/48/1/015001.
